# Chronic infection control relies on T cells with lower foreign antigen binding strength generated by N-nucleotide diversity

**DOI:** 10.1371/journal.pbio.3002465

**Published:** 2024-02-01

**Authors:** Hassan Jamaleddine, Dakota Rogers, Geneviève Perreault, Jérémy Postat, Dhanesh Patel, Judith N. Mandl, Anmar Khadra

**Affiliations:** 1 Department of Physiology, McGill University, Montreal, Quebec, Canada; 2 McGill University Research Centre on Complex Traits, Montreal, Quebec, Canada; 3 Department of Microbiology and Immunology, McGill University, Montreal, Quebec, Canada; Children’s Hospital of Philadelphia and The University of Pennsylvania School of Medicine, UNITED STATES

## Abstract

The breadth of pathogens to which T cells can respond is determined by the T cell receptors (TCRs) present in an individual’s repertoire. Although more than 90% of the sequence diversity among TCRs is generated by terminal deoxynucleotidyl transferase (TdT)-mediated N-nucleotide addition during V(D)J recombination, the benefit of TdT-altered TCRs remains unclear. Here, we computationally and experimentally investigated whether TCRs with higher N-nucleotide diversity via TdT make distinct contributions to acute or chronic pathogen control specifically through the inclusion of TCRs with lower antigen binding strengths (i.e., lower reactivity to peptide-major histocompatibility complex (pMHC)). When T cells with high pMHC reactivity have a greater propensity to become functionally exhausted than those of low pMHC reactivity, our computational model predicts a shift toward T cells with low pMHC reactivity over time during chronic, but not acute, infections. This TCR-affinity shift is critical, as the elimination of T cells with lower pMHC reactivity *in silico* substantially increased the time to clear a chronic infection, while acute infection control remained largely unchanged. Corroborating an affinity-centric benefit for TCR diversification via TdT, we found evidence that TdT-deficient TCR repertoires possess fewer T cells with weaker pMHC binding strengths *in vivo* and showed that TdT-deficient mice infected with a chronic, but not an acute, viral pathogen led to protracted viral clearance. In contrast, in the case of a chronic fungal pathogen where T cells fail to clear the infection, both our computational model and experimental data showed that TdT-diversified TCR repertoires conferred no additional protection to the hosts. Taken together, our *in silico* and *in vivo* data suggest that TdT-mediated TCR diversity is of particular benefit for the eventual resolution of prolonged pathogen replication through the inclusion of TCRs with lower foreign antigen binding strengths.

## Abstract 2

Although more than 90% of the sequence diversity among T cell receptors (TCRs) is generated by terminal deoxynucleotidyl transferase (TdT)-mediated nucleotide addition, the benefit of TdT-altered TCRs remains unclear. This study shows that low-affinity T cells generated by TdT are important for curtailing chronic pathogen replication.

## Introduction

The generation of lymphocyte receptor diversity is a key feature of adaptive immunity [1,2]. For T cells, this diversity is established by somatic recombination of the V(D)J gene segments that constitute the α- and β-chains of the T cell receptor (TCR) [2]. The T cell response to any one pathogen consists of a number of T cell clonotypes, each expanded from a rare antigen-specific T cell defined by the unique TCR they express. Every T cell clonotype in a given antipathogen response recognizes the same or different antigens in the form of peptides presented by major histocompatibility complexes (pMHCs) [3], and T cell clonotypes can differ in their ligand binding strengths by several orders of magnitude [4,5]. Recent evidence suggests that heterogeneity among responding T cells in their TCR binding affinity to pMHC, henceforth referred to as pMHC reactivity, correlates with important differences in effector function. For instance, pMHC binding strength in CD4^+^ T cells has been shown to impact early effector lineage differentiation [6–8], while among CD8^+^ T cells, it correlates with both their ability to induce target cell lysis and proliferative capacity and also impacts memory T cell development [5]. However, to what extent the pMHC-reactivity distribution of the T cells that constitute a given response affects how quickly a pathogen can be cleared remains incompletely understood.

Experimental techniques are currently limited in their ability to comprehensively study the temporal evolution of T cell clonotype frequencies with distinct pMHC reactivities that make up the antigen-specific response. One common method of identifying antigen-specific T cells is by pMHC tetramers, which primarily tag clonotypes on the higher end of the pMHC-reactivity spectrum, while missing most low affinity T cells [4]. Employing pMHC tetramers also requires a priori knowledge of the epitope recognized by the T cell population being investigated—tracking only the T cells that are specific to one epitope rather than the entire population of responding T cells. Two-dimensional (2D) binding assays measuring TCR-ligand binding affinity similarly rely on knowing the specific epitope recognized by the T cells under study [9]. Tracking T cell responses by focusing on only a subset of epitope-specific T cells can therefore introduce biases and disregards the contribution of the remaining pathogen-specific T cell population. Complementing experimental results with a theoretical framework that accounts for pMHC reactivity in a T cell repertoire is thus a useful approach to obtaining a clearer understanding of the mechanisms impacting pMHC-reactivity profiles and, consequently, T cell responses to infection.

A critical contribution to the diversification of the T cell repertoire in all jawed vertebrates is made by a DNA polymerase, called terminal deoxynucleotidyl transferase (TdT), that adds nontemplated nucleotides to the V(D)J junctions in αβTCRs [2,10–12], enhancing TCR repertoire diversity approximately 10-fold from the germline recombinatorial diversity alone [13–15]. However, the benefit of the N-diversification mediated by TdT has remained elusive given that TdT-knockout (KO) mice have shown no increased susceptibility to infection, nor any detectable impairment in their response to challenge with an acute pathogen [10,16]. Interestingly, the genetic sequence and structure of TdT is highly conserved across vertebrates [17,18], suggesting a hitherto unclear evolutionary benefit for its mechanism of action. One hypothesis proposed is that TdT introduces TCRs that, on average, possess lower reactivity to foreign pMHC [19]. Indeed, TdT KO T cells are more efficiently positively selected in the thymus, suggesting they may have inherently greater pMHC affinity [20]. Moreover, upon influenza A virus infection, the HA_518_ epitope-specific CD8^+^ splenic T cells from TdT KO mice were about 10 times more sensitive to antigenic stimulation as measured by IFNγ production than epitope-specific CD8^+^ T cells from wild-type mice, again consistent with the idea that TdT-independent TCRs have higher ligand affinity [21]. Importantly, during chronic antigen stimulation in infection and cancer mouse models alike, T cells with higher pMHC-reactivity have been shown to be more prone to exhaustion, whereby their cytokine production and contribution to pathogen or tumor control is substantially impaired compared to their low-affinity counterparts [22–24]. Thus, TdT-dependent TCRs may confer an advantage during chronic infections if the more germline TCRs with higher pMHC reactivity are more likely to become exhausted and ineffective.

Here, we sought to generate a theoretical framework to examine the role of heterogeneity in T cell reactivity to foreign pMHC in the clearance of an acute or chronic pathogen. To predict the impact of a TdT-deficient T cell repertoire on infection outcomes, we developed and implemented a computational model capturing the kinetics of both acute and chronic pathogen replication that also explicitly considered the evolution of TCR affinity distributions during infection. Our simulations showed that, during chronic infection, there was a decrease in the average pMHC reactivity of the antigen-specific T cell population. Importantly, our computational model suggested that when the TCR repertoire lacked T cells with lower pMHC reactivity, pathogen clearance during chronic, but not acute, infection was delayed. In line with these *in silico* predictions, chronic lymphocytic choriomeningitis virus (LCMV) infection of mice with TdT KO T cells led to more protracted viral replication and a significant delay in viral clearance. In contrast, during chronic infection with *Cryptococcus neoformans* where T cells fail to clear the pathogen, both our computational model and experimental data instead showed that TdT-diversified TCR repertoires conferred no additional protection. Taken together, our model simulations and experimental results support the notion that one evolutionary benefit of TdT may be to improve chronic pathogen clearance by increasing the frequency of T cells with lower antigen-specific pMHC reactivity.

## Results

### Computational T cell model captures kinetics of pathogen load for both acute and chronic infection

**Fig 1:**
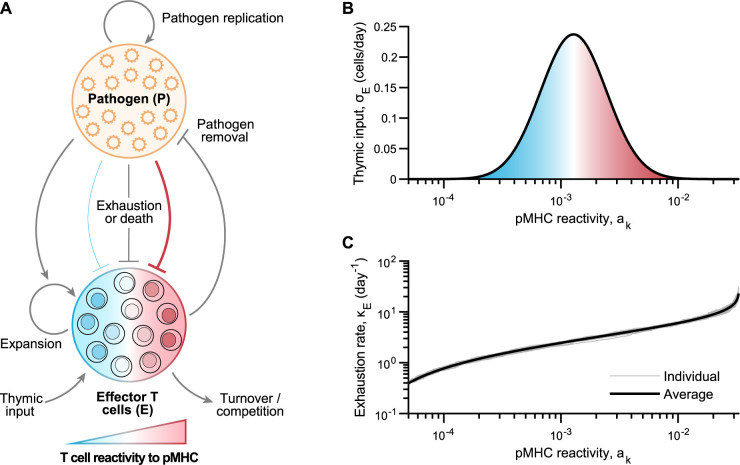
Implementing a computational population model defining the dynamics of pathogen replication and responding T cell clonotypes. (**A**) Model scheme illustrating model variables and their interactions. The model is described by a system of integro-differential equations that govern the rates of change of the 2 key players, namely, pathogen loads (*P*) and the effector T cells (*E*), which are specific for pathogen-derived antigens in the form of pMHC. The effector T cell population, encompassing the complete collection of pathogen-specific CD4^+^ and CD8^+^ T cells, are defined on a continuum according to their overall reactivity to pMHC (*a*_*k*_), i.e., the strength of the overall TCR–pMHC interactions (the shade from blue to red represents the reactivity continuum of T cells to pMHC that ranges from low to high TCR affinities, respectively). Pathogen load is subject to replication as well as negative regulation by effector T cells. Effector T cells expand upon pathogenic exposure, with a constant low-level thymic input, natural turnover, and intercellular competition. Pathogen persistence promotes T cell exhaustion and/or activation-induced cell death, with higher pMHC-reactivity T cells more susceptible to these than their low pMHC-reactivity counterparts. For simplicity, we (i) left out the role of other players such as antigen-presenting cells and B cells from the model in order to focus on the specific role of pMHC reactivity in defining dynamics, and (ii) did not explicitly distinguish between the action of CD4^+^ and CD8^+^ T cells. (**B, C**) Functions depicting pMHC reactivity-dependent parameter values, wherein thymic input (**B**), *σ*_*E*_ = *f*(*a*_*k*_), mimics the shape of a theoretical log-normal distribution, and T cell exhaustion rate (**C**), *κ*_*E*_, is determined by sampling from a shifted exponential distribution of a set mean and sorted in ascending order by assigning smaller depletion rates to T cells of low pMHC-reactivity, and vice versa, producing variability between model simulation runs (shown by gray traces obtained from 25 individual model simulations). See Methods to access code used to produce model simulations in this figure. pMHC, peptide-major histocompatibility complex.

In order to compare the temporal evolution of pMHC reactivities of responding T cell clonotypes during acute and chronic infections, we first needed a simple computational model that could recapitulate the kinetics of both rapidly cleared and prolonged pathogen replication. We developed a model that could replicate the serum viral loads obtained upon infection with 2 strains of LCMV that differ by only 3 coding point mutations, Armstrong (LCMV-Arm) and Clone 13 (LCMV-Cl13), and produce the time course of acute and chronic viral loads in the host, respectively [22,25,26]. Importantly, the single amino acid mutations between LCMV-Arm and LCMV-Cl13 do not occur in peptide segments from which known T cell–specific pMHC epitopes are derived [27,28], and, thus, different infection outcomes are governed exclusively by viral replication dynamics within infected cells [25,27]. Our model considered key interactions between the pathogen load (*P*) and the pMHC-reactivity continuum of all responding effector CD4^+^ and CD8^+^ T cells (*E*) (**Fig 1A**). The dynamics of *P* and *E* depend on the rates of pathogen replication, T cell proliferation and expansion upon antigen encounter, thymic input, homeostatic T cell turnover, and pathogen-induced exhaustion and/or cell death (**Fig 1A**). To incorporate pMHC reactivity explicitly into our model, we defined the thymic input (i.e., the source of antigen-specific precursors arising from the thymus) into the effector T cell pool, *σ*_*E*_, to be a function of pMHC reactivity (denoted *a*_*k*_; **Fig 1B**), a parameter proportional to the strength of TCR signaling (refer to Methods) whereby *a*_*k*_ represents how likely it is that a T cell will proliferate upon antigenic stimulation [29]. Consistent with previous studies, we assumed that the strength of negative regulatory mechanisms (such as T cell exhaustion and activation-induced cell death) is positively correlated with the pMHC reactivity of a given T cell [22–24]. Variability in model outcomes is generated by randomization of this T cell exhaustion rate, which is selected from a shifted exponential distribution and sorted in increasing order as a function of pMHC reactivity (with a mean determined by model fitting, see Methods) (**Fig 1C**).

**Fig 2:**
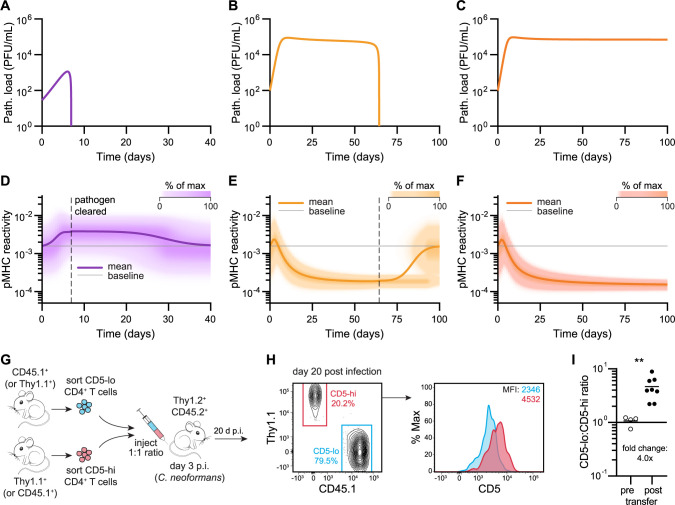
T cell pMHC reactivity evolution over time during a T cell response differs between acute and chronic infections. (**A-F**) Representative model simulations during an acute infection (**A**, **D**), a chronic infection that is eventually cleared (**B**, **E**), or a persistent chronic infection (**C**, **F**) showing time series simulations of pathogen loads (**A**-**C**) and heat maps representing the relative proportion of T cells across pMHC reactivities (**D**-**F**). Overlayed traces represent the mean pMHC-reactivity value in time, with the baseline value represented in gray. Dotted black lines (**D**, **E**) denote the time at which the pathogen is cleared. (**G**) Schematic of experimental approach illustrating adoptive cell transfer of a 1:1 ratio of CD5^lo^ and CD5^hi^ naïve CD4^+^ T cells (either Thy1.1^+^ or CD45.1^+^) into congenic CD45.2^+^ Thy1.2^+^ recipient mice infected 3 days prior with 5 × 10^3^ CFU of chronic *C*. *neoformans*. (**H**) Representative flow cytometry plot of CD5^lo^ CD45.1^+^ and CD5^hi^ Thy1.1^+^ transferred T cell populations 20 days post-infection with CD5 expression levels shown as a histogram and MFIs indicated in blue and red text. (**I**) Ratio of transferred CD5^lo^ to CD5^hi^ T cells, preinjection or 20 days post-infection with *C*. *neoformans*. ** *P* < 0.01 computed using a two-tailed Wilcoxon rank sum test, data from 2 independent experiments (*n* = 8 mice). The experimental data underlying this figure can be found in **S1 Data**. See Methods to access code used to produce model simulations in this figure. CFU, colony-forming unit; MFI, mean fluorescent intensity; pMHC, peptide-major histocompatibility complex.

Model parameters (**S1 Table**) were obtained by fitting simulated pathogen loads to serum data of LCMV infections from Wherry and colleagues’ study [22] using a genetic algorithm (**S1A** and **S1B Fig**). Since previous work has shown that LCMV-Cl13 replicates more rapidly in infected cells than LCMV-Arm [25], we set the replication rate (denoted *r*_*p*_) for our simulated chronic viral infection to be higher than for the acute infection, while keeping all other parameters consistent between the 2 conditions. By modulating only the pathogen replication rate and initial pathogen loads, we produced time courses that qualitatively matched both LCMV-Arm and LCMV-Cl13 replication dynamics (**Figs 2A, 2B, S1A and S1B**). In our simulations, the acute viral load peaked at 6.0 days post-infection, followed by rapid clearance around 6.9 days post-infection based on 100 trial simulations (**Figs 2A** and **S1C**). In contrast, increasing the viral replication rate to simulate chronic viral replication resulted in prolonged infections with a median time to clearance of 64 days (based on 100 trial simulations) (**Figs 2B** and **S1C**). Given that many chronic pathogens differ from LCMV-Cl13 in that they are never cleared (i.e., they persist indefinitely within the host and may even lead to host death, as is the case with fungal *C*. *neoformans* [30]), we also simulated the model with a larger pathogen replication rate to additionally account for the dynamics of such pathogens. Further increasing pathogen replication in our computational model led to elevated pathogen loads in our simulations that persisted indefinitely (**Fig 2C**). Thus, our computational model was able to reproduce pathogen loads characteristic of both acute and chronic infections, with the latter being either eventually cleared or persisting indefinitely (or at least until the host succumbs to infection).

### Chronic infection skews responding T cell clonotypes toward lower pMHC reactivities

Having developed our *in silico* model of acute and chronic pathogen infection, we next asked how the pMHC-reactivity profile of the antigen-specific T cell population evolved over time in each case. When we simulated effector T cell responses to acute infection, we found that the pMHC-reactivity profile of T cells shifted toward a higher pMHC-reactivity mean until peak pathogen replication was reached, followed by a gradual return to baseline after the infection was resolved (**Fig 2D** and **S1 Movie**). Of note, the return of the pMHC-reactivity mean value to the pre-infection baseline was a result of omitting a memory T cell compartment from the model, since our focus was on the effector phase of the T cell response. The shift we observed in the pMHC-reactivity profile during acute infection agrees with previous experimental studies showing that T cells with greater pMHC reactivity expand to large numbers more readily upon antigenic stimulation [31–34].

Next, we investigated whether the dynamics of pMHC reactivity among responding T cell clonotypes differed during chronic infection. In contrast to acute infection, the pMHC-reactivity distribution peaked during the early phases of chronic infection but was followed by a substantial shift toward lower pMHC reactivities, before returning to baseline after infection clearance (**Fig 2E** and **S1 Movie**). This indicated that, as more T cells of high pMHC reactivity became functionally exhausted due to chronic antigen stimulation (and their relative contribution to the active effector T cell pool was therefore reduced), T cells of progressively lower reactivities to pMHC gradually predominated among responding T cell clonotypes. We observed a similar shift toward T cells with lower pMHC reactivity in persistent chronic infection (**Fig 2F**), with the main difference being a lack of the return to baseline since T cells fail to clear the infection.

To further investigate whether our computational model was consistent with experimental data, at least for CD4^+^ T cells, we used a previously identified surface marker proxy for pMHC reactivity, CD5, whose expression level on CD4^+^ T cells is a read-out of self-antigen reactivity, correlates with foreign pMHC binding strength as measured by tetramer fluorescent intensity, and is maintained post activation [34–36]. We sorted naïve CD4^+^ T cells on the 20% CD5^lo^ and CD5^hi^ cells, as previously described [34,36], mixed the 2 sorted populations at a 1:1 ratio (identified by congenic markers, CD45.1 or Thy1.1), and adoptively transferred the mix to CD45.2^+^ Thy1.2^+^ recipient mice that were infected 3 days earlier with *C*. *neoformans* (**Figs 2G** and **S1D–S1F**). In contrast to what was previously described during acute infections, where the CD5^hi^ CD4^+^ T cells predominated the response on day 8 post-infection [34], in the later stage of the anti-*C*. *neoformans* CD4^+^ T cell response in the lung, the CD5^lo^ CD4^+^ T cells outnumbered CD5^hi^ CD4^+^ T cells 4-fold (**Fig 2H and 2I**). To ask whether these results hold for another chronic pathogen, we adoptively transferred either sorted CD5^hi^ or CD5^lo^ CD4^+^ T cell populations into recipient (CD45.2^+^) mice that were subsequently infected with LCMV-Cl13 and assessed the proportions of transferred CD5^hi^ or CD5^lo^ cells 59 days post-transfer (**S1G Fig**). Consistent with our *C*. *neoformans* infection results, we observed an overrepresentation of CD5^lo^ CD4^+^ T cells relative to their CD5^hi^ counterparts (albeit nonsignificant) (**S1H and S1I Fig**). Importantly, CD5 expression levels on LCMV-specific CD4^+^ T cells have previously been shown to relate to their binding strength to LCMV-derived foreign pMHC using GP66:I-A^b^ tetramer staining [34]. Thus, while CD5 levels in CD4^+^ T cells correspond primarily to their reactivity for *self*-pMHC, in this context, a dominance in CD5^lo^ CD4^+^ T cells in chronic infection is consistent with the shift toward T cells with lower *foreign-*pMHC reactivity (as predicted by the computational model).

In summary, our modeling results suggest that the temporal evolution of pMHC reactivities of T cells contributing to the response to an acute compared to a chronic infection is distinct. During chronic infection, the antigen-specific T cells of lower pMHC reactivities predominate, whereas T cells of greater pMHC reactivity predominate during an acute infection. Our experimental data from CD4^+^ T cells corroborated this result and are consistent with evidence from other experimental studies of both antigen-specific CD4^+^ and CD8^+^ T cells indicating that T cells with lower pMHC reactivity predominate during chronic infection [37–39].

### The distribution of pMHC reactivities of responding T cells impacts time to infection clearance

**Fig 3:**
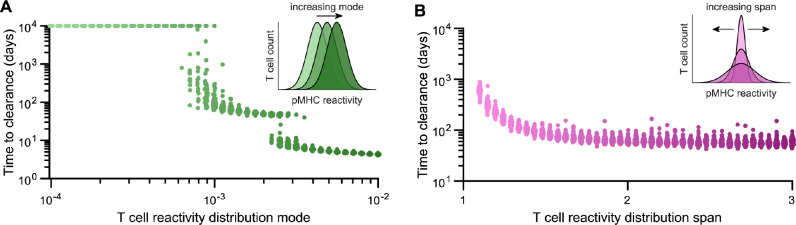
Mode and span of the naïve T cell pMHC-reactivity profile impact pathogen clearance times. (**A, B**) Time required to clear a replicating pathogen *in silico* when varying the mode (**A**) and the span (**B**) of the initial T cell count as a function of pMHC reactivity. Each data point shows the result of a single simulation trial for a given, randomized set of parameters for T cell exhaustion (*κ*_*E*_) as determined by the relation defined in Fig 1C. Insets schematically indicate how the T cell count of the starting (pathogen-free/baseline) configuration, as a function of pMHC reactivity, is altered by increasing its mode (**A**) or span (**B**). The distribution mode and span shown are equivalent to $e^{-(\mu +\sigma^{2})}$ and *e*^*σ*^, respectively, where *μ* and *σ* are, respectively, the mean and standard deviation of the log-normal pMHC reactivity function defining thymic input (for full details, see **S1 Text**). See Methods to access code used to produce model simulations in this figure. pMHC, peptide-major histocompatibility complex.

Having predicted differential evolution in the pMHC reactivity of effector T cells depending on the duration of infection using our computational model, we next wanted to determine whether the reverse is true, i.e., whether altering the pMHC-reactivity profile of the antigen-specific T cell population would lead to changes in the duration of pathogen replication upon infection. To accomplish this, we targeted model parameters affecting either the mode or the span of the T cell pMHC-reactivity distribution to investigate how perturbing these 2 parameters impact the T cell response to a pathogen. In these simulations, we maintained the pathogen replication rate at a constant value obtained from fitting the computational model to LCMV-Cl13 viral loads and used the time at which pathogen burden returned to 0 post-infection as a measure to assess (i) class of infection (i.e., acute versus chronic) and (ii) effectiveness of the T cell response in clearing infection. Gradually varying the mode of the pMHC-reactivity profile over 40 logarithmically spaced values between 10^−4^ and 10^−2^ and running 50 independent simulations per mode value revealed 3 distinct clusters with different durations of infection (**Fig 3A**). Variability between different runs of the model arises from the randomization of the exhaustion rate, *κ*_*E*_, in each simulation, as described previously (**Fig 1C**). When the mode of antigen-specific pMHC reactivities of the T cell repertoire was too low (less than 7 × 10^−4^), the infection persisted indefinitely, indicating that T cells failed to clear the pathogen. At intermediate values of pMHC reactivity (between 7 × 10^−4^ and 2 × 10^−3^), the time to clearance was around 100 days, comparable to chronic LCMV-Cl13 infection. When reactivity to foreign pMHC was high, the infection was cleared within 10 days, representative of an acute infection (**Fig 3A**). Varying the span of the pMHC reactivity distribution also affected pathogen replication kinetics, with narrow pMHC reactivity distributions exhibiting impaired chronic pathogen clearance compared to T cell repertoires with broader distributions (**Fig 3B**).

Taken together, our simulation results demonstrate that the pMHC-reactivity profile of responding T cells during infection has a pronounced effect on determining infection duration, with outcomes ranging from rapid pathogen clearance to the complete failure of the T cell response to resolve the infection. Of note, rather than observing gradual shifts in the time to clearance with *in silico* manipulations of the pMHC-reactivity profile, we found sharp jumps between different infection clearance times (namely, from acute to chronic, and chronic to indefinitely persistent). By reducing the computational model to a one-clone, 2D system of ordinary differential equations and performing bifurcation analysis (**S2 Fig**), we found that distinct solution trajectories through state space are responsible for producing separate clusters of infection durations. Importantly, analysis of the one-clone model showed that, exclusively in the case of chronic pathogen load, the evolution of the T cell profile toward lower pMHC reactivities is responsible for the eventual resolution of chronic infection (**S2 Fig** and **S2 Movie**, see **S1 Text**).

### Computational model predicts a delay in chronic infection clearance in the absence of T cells with low pMHC-reactivity

**Fig 4:**
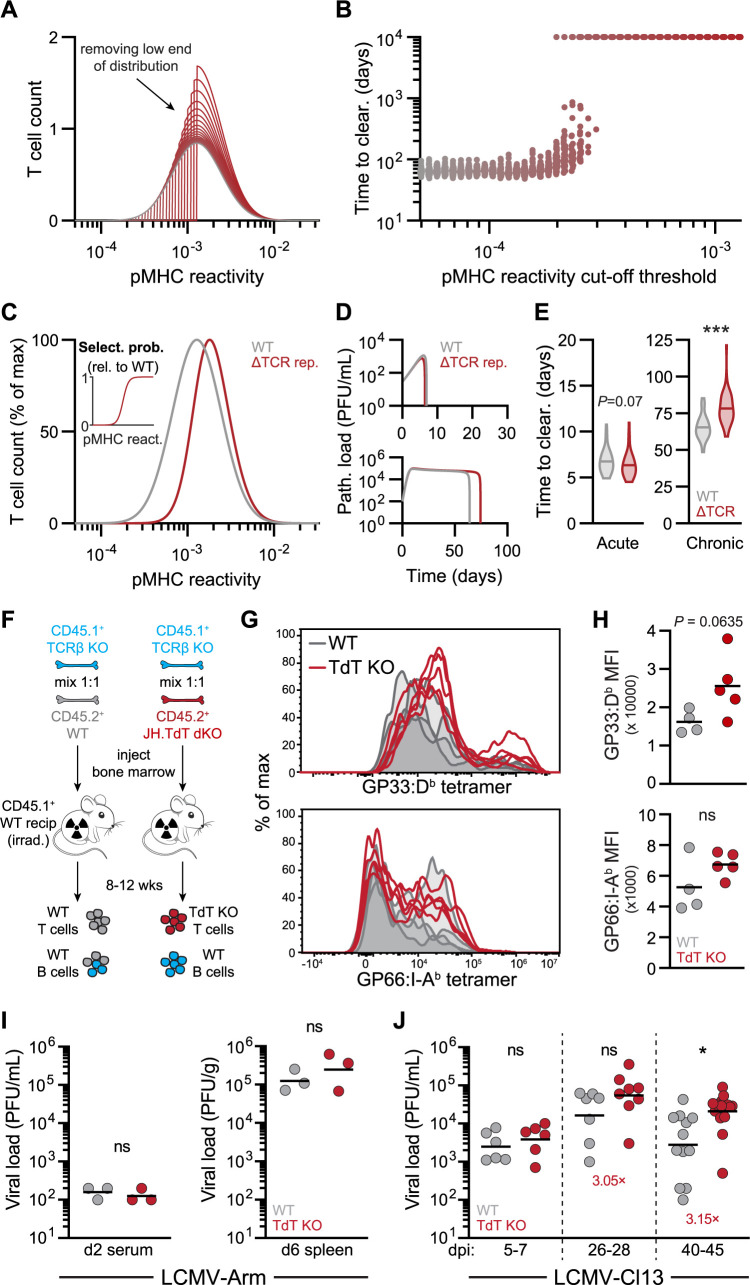
TdT deficiency in T cells *in silico* and *in vivo* impairs control of chronic, but not acute, infections. (**A**) Distributions of T cells as a function of pMHC reactivity obtained by successively removing low-affinity T cells using different cutoff thresholds from the model’s starting configuration, while keeping the total number of T cells conserved, until only the upper half of the distribution remained. These starting configurations were generated by setting all values of the thymus input, *σ*_*E*_, for T cells below a given pMHC-reactivity threshold to 0. (**B**) Time to pathogen clearance obtained by increasing the cutoff threshold for T cell reactivity to pMHC corresponding to the configurations shown in (**A**). For each cutoff threshold, 50 simulation trials were performed as described in Fig 3. Pathogen replication rate was fixed to the chronic setting (*r*_*P*_ = 1.22 day^−1^). (**C**) Theoretical T cell pMHC-reactivity configuration of an altered TCR repertoire (denoted ΔTCR repertoire) deficient in low-affinity T cells relative to the WT configuration. The ΔTCR repertoire was assumed to have a lower thymic selection probability (relative to WT repertoire), implying a lower value of the thymic input parameter, *σ*_*E*_, for T cells with low pMHC reactivity (see Methods). Inset: probability of selection, relative to the WT repertoire, for T cells of the ΔTCR repertoire, with fewer T cells of low reactivity to pMHC being sourced by thymic selection. (**D**) Model simulations comparing representative pathogen load traces of WT (gray) or ΔTCR repertoire (red) systems during acute (top) or chronic (bottom) infection. (**E**) Time to clearance of acute (left) or chronic (right) infections for 50 model simulations (log-values of initial pathogen loads randomized to ±10% of the values in S1 Table) from WT and ΔTCR repertoire systems. Horizontal lines indicate mean values. (**F**) Generation of BM chimeric mice possessing WT B cells, and either WT T cells (reconstitution of irradiated mice with 1:1 ratio of BM from WT mice and TCRβ KO mice) or TdT KO T cells (reconstitution with 1:1 ratio of BM from JH and TdT double KO mice and TCRβ KO mice). (**G, H**) Histogram overlays of MHC class I D^b^ GP33 and MHC class II I-A^b^ GP66 tetramer fluorescence (**G**) and summary of tetramer MFI (**H**) of activated (CD44^hi^) tetramer^+^ CD8^+^ and CD4^+^ T cells, *n* = 4–5 chimeras. Data for GP66:I-A^b^ tetramer staining are representative of 4 independent experiments. (**I**) LCMV-Arm viral loads in the serum (left) and spleen (right) of mice with WT or TdT KO T cells measured at 2 or 6 days post-infection, respectively (*n* = 3 mice per group). (**J**) LCMV-Cl13 viral loads in the serum of mice with WT or TdT KO T cells in early (5–7 days post-infection), mid (26–28 days post-infection), or late (40–45 days post-infection) stages of viral replication (*n* = 6–18 mice per group). Fold-changes of viral loads in mice with TdT-deficient T cells, relative to WT, are indicated. *P* values indicated were computed using Wilcoxon rank sum test (**E**), Mann–Whitney test (**H**), unpaired *t* test (**I**), and Kruskal–Wallis test (**J**). ns = not significant, * *P* < 0.05, *** *P* <0.001. The experimental data underlying this figure can be found in **S1 Data**. See Methods to access code used to produce model simulations in this figure. BM, bone marrow; KO, knockout; LCMV, lymphocytic choriomeningitis virus; MFI, mean fluorescent intensity; pMHC, peptide-major histocompatibility complex; TCR, T cell receptor; TdT, terminal deoxynucleotidyl transferase; WT, wild type.

Our simulations showed that an increased contribution from antigen-specific T cell clonotypes with low pMHC reactivity could be important in clearing chronic, but not acute, infections. Thus, we next investigated whether populating a TCR repertoire exclusively with higher pMHC reactivity T cells would affect the clearance of either an acute or a chronic infection in silico. We tackled this question in 2 ways. First, in our simulations, we removed antigen-specific T cells with lower pMHC reactivity by eliminating all T cells possessing a pMHC reactivity value below a predefined cutoff threshold (without altering the total number of responding T cells) and then progressively increased this threshold to remove up to half of the pMHC reactivity distribution (**Fig 4A**). As with our previous analysis, in these simulations, we maintained the pathogen replication rate at a constant value obtained from fitting the model to LCMV-Cl13 viral loads. Performing this analysis demonstrated that impeding the shift toward lower TCR affinities during a chronic infection by progressively removing T cells with lower pMHC reactivity in this manner dramatically prolonged the time to clearance for the chronic infection cluster, with higher cutoff thresholds leading to failure to clear the chronic pathogen altogether (**Fig 4B**). Interestingly, repeating this analysis while decreasing the pathogen replication rate from 1.22 day^−1^ to 1.17 day^−1^ revealed that high cutoff thresholds could result in rapid pathogen clearance owing to the greater number of only high pMHC reactivity T cells; this led to the formation of an acute infection cluster (**S3A and S3B Fig**). This result is consistent with Fig 3A, where higher pMHC-reactivity modes can prevent infection chronicity altogether. When the number of high pMHC reactivity T cells was kept unchanged, the acute cluster did not form at high values of the cutoff threshold (**S3C and S3D Fig**).

While gradually removing all effector T cells below a certain pMHC-reactivity threshold allowed us to investigate the distinct roles of T cells with low versus high pMHC reactivity, we next designed a second approach in which we also modified our model to simulate a more biologically plausible change in the pMHC reactivity profile. In this approach, we defined an altered TCR repertoire (ΔTCR rep.), wherein the probability that low-reactivity T cells selected for in the thymus was reduced, while the selection probability of high-reactivity T cells was left relatively unchanged (**Fig 4C**). We found that this ΔTCR rep. hardly altered replication kinetics of an acute pathogen simulated using parameters obtained from fitting the computational model to LCMV-Arm viral loads (**Fig 4D**, top), with little effect on the time taken to clear it (**Fig 4E**, left). However, testing the ΔTCR rep. model with a chronic pathogen (using parameters obtained from fitting to LCMV-Cl13 viral loads) revealed that chronic infection clearance was impaired (**Fig 4D**, bottom), and time to clearance was significantly longer (**Fig 4E**, right). In summary, altering the pMHC-reactivity profiles of responding T cells by introducing reductions in T cells with low pMHC-reactivity showed that modulating only the TCR repertoire pMHC reactivity led to impaired control of chronic, but not acute, infections.

### Absence of TdT-dependent TCRs *in vivo* impairs chronic pathogen clearance consistent with a deficiency in T cells with lower antigen binding strengths

The hypothesis that N-diversity mediated by TdT, which accounts for 90% to 95% of the TCR repertoire diversity [11], disproportionately generates lower-affinity TCRs [19] has been difficult to address experimentally without a specific prediction of the type of infection that these low-affinity TCRs are important for with regard to curtailing pathogen replication. Based on our modeling results suggesting that a TCR repertoire deficient in T cells with low pMHC reactivity would lead to an impaired effector T cell response during chronic infection, we would similarly expect a TdT-deficient repertoire to produce delayed chronic, but not acute, pathogen clearance. However, before testing this prediction *in vivo* in the context of antigenic challenge, we first investigated whether TdT-deficient T cells might possess higher self-pMHC reactivity as read out by CD5 expression levels (given our earlier results with sorted CD5 populations; **Fig 2G–2I**). To test this, we generated mixed bone marrow (BM) chimeras where BM from TdT KO and WT donors were mixed at a 1:1 ratio and transferred into lethally irradiated, congenic (Thy 1.1^+^) mice (**S4A Fig**). In line with the notion that TdT KO T cells possess higher affinity for pMHC, we observed consistently higher CD5 levels in pre-selection double-positive (DP) TdT KO thymocytes compared to their WT counterparts present within the same mouse (**S4B Fig**, left). Intriguingly, this robust difference in DP pre-selection CD5 levels disappears at the single-positive (SP) CD4^+^ and CD8^+^ stage after the thymocytes undergo selection (**S4B Fig**, right), possibly due to the manner in which CD5 levels are mapped from the DP to the SP stage together with the imposition of lower and upper bounds set by positive and negative selection, respectively. We next compared the ratios of TdT KO to WT SP CD4^+^ and CD8^+^ thymocytes relative to the number of pre-selection DP thymocytes, to ask whether there was any indication that TdT KO T cells are more efficiently positively selected as previously suggested [20]. Indeed, we found a significant overrepresentation of TdT KO compared to WT SP thymocytes (**S4C Fig**), in line with the notion that TdT KO T cells are enriched for TCRs with higher affinity for self-pMHC that are more efficiently positively selected as a result.

We next asked whether TdT KO T cells bind their cognate foreign antigen more strongly in the context of infection and whether these T cells are thus more prone to exhaustion during chronic antigenic challenge (a key assumption of our computational model). Since TdT also inserts nontemplated nucleotides into the B cell receptor during B cell development [40], and the altered B cell receptor repertoire might therefore impact viral clearance, we restricted the TdT deficiency to the T cell compartment, with B cells expressing normal levels of TdT. To do so, we generated BM chimeras (**Fig 4F**) whereby TCRβ KO BM was mixed 1:1 with either WT BM (leading to development of WT B and T cells) or with BM obtained from TdT and JH double KO mice (leading to the development of WT B cells and TdT KO T cells). We verified that our BM reconstitutions led to a 1:1 ratio of hematopoietic cell development from each of the donors, identified using congenic markers (**S4D Fig**). We then infected these chimeras with LCMV-Cl13 and stained activated T cells with tetramers for the immunodominant CD4^+^ and CD8^+^ epitopes (GP66:I-A^b^ and GP33:D^b^, respectively) 45 days post-infection. Indeed, we found that staining intensities of tetramer-positive TdT KO T cells with both GP33:D^b^ and, to a lesser extent, GP66:I-A^b^ tetramers were higher than for WT T cells, suggesting higher antigen binding capacity on average of TdT KO T cells (**Fig 4G and 4H**) at least for these particular epitopes. Furthermore, consistent with the model assumption that high-affinity T cells are more prone to functional exhaustion upon chronic infection, we also saw higher expression levels of exhaustion marker PD1 in both tetramer-positive and total activated (CD44^hi^) TdT KO T cell populations for CD4^+^ T cells (**S4E and S4F Fig**), though we did not observe a difference in PD1 expression levels in CD8^+^ T cells. Nonetheless, these data provide support for the notion that TdT preferentially leads to the generation of lower-affinity T cells that may be, at least in the case of CD4^+^ T cells, more resistant to antigen-induced exhaustion.

To test the model prediction that TdT-dependent TCR repertoire diversification would improve chronic pathogen control for a pathogen that is ultimately cleared, we next measured viral burdens in mice infected with either acute LCMV-Arm or chronic LCMV-Cl13. In line with previous results from full TdT KO mice in response to acute LCMV infection [16], we observed no differences in the viral load following LCMV-Arm infection between the WT T cell and TdT KO T cell groups, both 2 days post-infection in the serum and 6 days post-infection in the total spleen homogenate (**Fig 4I**). In contrast, mice with TdT KO T cells had a significantly higher viral load (3.15-fold increase) during the chronic phase (days 40 to 45) following infection with LCMV-Cl13 (**Fig 4J**). These data were qualitatively consistent with computational model simulations of LCMV-Cl13 pathogen loads at equivalent time points showing higher pathogen loads in the ΔTCR rep. model (i.e., in the absence of T cells with lower pMHC reactivity) specifically in the later stages of infection (**S4G Fig**). Importantly, these simulations also revealed a modest, albeit significant, reduction in pathogen loads early in LCMV-Cl13 infection owing to the increased proportion of higher-affinity T cells in the ΔTCR rep. model. While this difference could not be resolved in the experimental viral load data (**Fig 4J**), this is likely a result of the smaller sample sizes. Since irradiated BM recipients still retain some endogenous hematopoietic stem cells, we repeated the LCMV-Cl13 infection in BM chimeras using TCRβ KO recipient mice instead (i.e., mice lacking endogenous αβT cells) and obtained similar results where chimeras with TdT KO T cells had a 3.74-fold increase in viral load by day 41 post-infection (**S4H** and **S4I Fig**).

Previous experimental and theoretical studies alike have shown that limiting the breadth of the T cell response to infection, or introducing “gaps” in the TCR repertoire, can impede pathogen control [41–43]. We therefore wanted to investigate whether introducing similar gaps in the context of our pMHC-reactivity model could equally recapitulate the viral load data in mice with TdT KO T cells, demonstrating impaired chronic (but not acute) pathogen control. To test this, we constructed an alternative ΔTCR repertoire where we decreased the number of T cell clonotypes 10-fold, without affecting the overall affinity profile or the total population size of the precursor pool (**S5A Fig**). Interestingly, when we simulated both acute and chronic infections using this alternative ΔTCR repertoire configuration, we found no differences in pathogen clearance in either case (**S5B and S5C Fig**), inconsistent with what we saw in mice with TdT KO T cells (**Fig 4I and 4J**). Incidentally, we also tested whether decreasing T cell precursor frequencies 10-fold instead (evenly across all pMHC-reactivity values) might recapitulate our experimental results but again found a mismatch between the data and the simulations wherein acute, and not chronic, infection control seemed to be impaired relative to the WT configuration (**S5D–S5F Fig**). Thus, while our modeling does not rule out the possibility for additional, affinity-independent advantages of TCR repertoire diversification by TdT, when taken together, these results lend further support to the notion that TdT benefits hosts challenged with chronic infections and that it does so, at least in part, by generating T cells with lower affinity for foreign pMHC.

### Lack of TdT-dependent low-affinity TCRs has a protective effect against noncleared pathogens *in silico* and delays infection induced mortality *in vivo*

Taken together, our computational and experimental results thus far have suggested that TdT deficiency impairs clearance of a chronic pathogen through the omission of T cells with lower foreign pMHC reactivity. However, not all pathogens that produce chronic infections are cleared by the host T cell response; for example, *C*. *neoformans* continues to replicate until the host eventually succumbs to the infection. Therefore, we next investigated how the absence of TdT might impact the T cell response to, and consequently the control of, a persistent pathogen (i.e., a pathogen that is not cleared).

**Fig 5:**
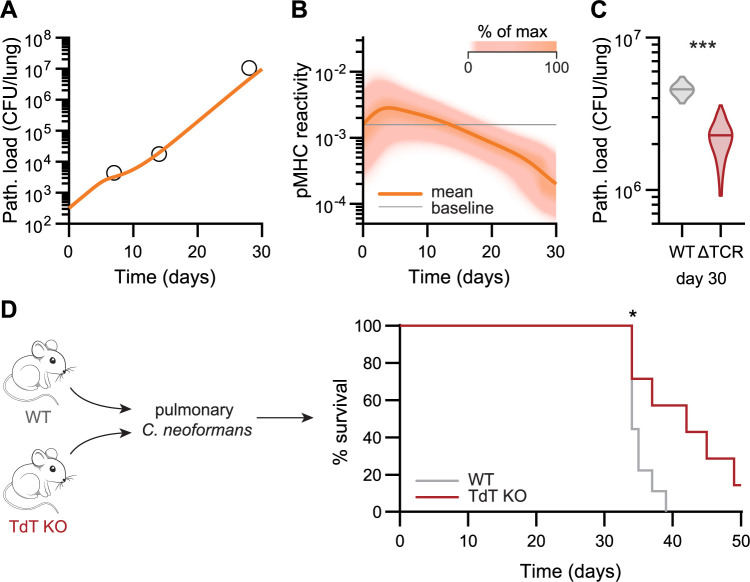
TdT deficiency delays persistent infection mortality. (**A, B**) Representative time series simulations of pathogen load (**A**) and heat maps representing the relative proportion of T cells across pMHC reactivities (**B**) obtained by retuning the computational model from Fig 1 to reproduce *C*. *neoformans* lung pathogen loads digitized from [44]. Simulations were generated by modifying initial pathogen load (*P*_0_ = 316 CFU/lung), pathogen replication rate (*r*_*P*_ = 0.4 day^-1^), carrying capacity (*P*_max_ = 10^8^ CFU/lung), and clearance rate by T cells (*κ*_*P*_ = 1.5 × 10^−3^ CFU lung^−1^ cell^−1^ day^−1^); all other parameters were held fixed at their default values listed in S1 Table. Overlayed traces represent the mean pMHC-reactivity value in time, with the baseline value represented in gray. (**C**) Simulated pathogen loads by day 30 post-infection in the model, using either the WT or the ΔTCR repertoire configurations as shown in Fig 4C. (**D**) Kaplan–Meyer survival plot of WT (*n* = 9) or TdT KO (*n* = 7) mice infected with *C*. *neoformans*. *P* values were determined by Wilcoxon rank sum test (**C**) or Mantel–Cox test (**D**). * *P* < 0.05, *** *P* < 0.001. The experimental data underlying this figure can be found in **S1 Data**. See Methods to access code used to produce model simulations in this figure. CFU, colony-forming unit; KO, knockout; pMHC, peptide-major histocompatibility complex; TdT, terminal deoxynucleotidyl transferase; WT, wild type.

We started by tuning the computational model to specifically capture the time course of a *C*. *neoformans* infection. This was done by digitizing published lung pathogen load data in mice (untreated cohort) [44], then modifying the pathogen replication rate, initial pathogen load, total carrying capacity, and clearance rate by effector T cells in the model (while keeping all other parameters unchanged) to reproduce the time course of *C*. *neoformans* pathogen loads (**Fig 5A**). Consistent with the more generalized simulations of a noncleared chronic pathogen (**Fig 2C**) and our experimental data showing an overrepresentation in CD5^lo^ T cells upon *C*. *neoformans* infection (**Fig 2I**), the computational model tuned to *C*. *neoformans* similarly predicts a shift toward low-affinity T cells in the chronic phase of the infection (**Fig 5B**).

Next, we tested the *in silico* effects of reducing the number of T cells with low foreign pMHC reactivity, to generate a testable prediction for *C*. *neoformans-*infected TdT KO compared to WT mice. Given that *C*. *neoformans* is not cleared, we could not use the time to clearance as a predicted parameter in this context. Instead, we compared predicted pathogen loads after 30 days post-infection with the WT versus the altered ΔTCR repertoire configuration (with a skew toward T cells with higher foreign antigen binding strengths in the latter; **Fig 4C**). Interestingly, in contrast to a chronic pathogen that is eventually cleared (**Fig 4D and 4E**), we observed that the ΔTCR repertoire now provided increased protection relative to the WT configuration in the computational model (**Fig 5C**), with lower average pathogen loads by day 30. In this case, since there is already limited control of pathogen replication during the chronic phase of the infection when low-affinity T cells become dominant (as evidenced by continued exponential growth late in infection; **Fig 5A**), the lack of these low-affinity T cells in the ΔTCR repertoire did not further impair the anti-pathogen response. However, there is also an increased proportion of high-affinity T cells in the ΔTCR repertoire; thus, in these simulations, T cells with higher foreign pMHC reactivity provided an additional level of control during the early stages of pathogen replication (as seen when simulating earlier time points of an LCMV-Cl13 infection; **S4G Fig**), hence a lower pathogen load early on that carried through to the chronic stage of infection.

To test whether TdT deficiency would therefore protect hosts from a non-cleared chronic pathogen *in vivo*, we infected either WT or TdT KO mice with *C*. *neoformans* and assessed survival. Indeed, consistent with our model predictions, we found that TdT KO mice survived significantly longer upon infection than WT mice (**Fig 5D**). Notably, while the TdT KO mice also have altered B cell receptor repertoires (unlike the BM chimeras used in the LCMV experiments), B cell–dependent antibody production was shown to have little effect on *C*. *neoformans* pathogen loads or survival in systemically infected mice [45]. Thus, the observed difference in *C*. *neoformans*-induced mortality in our data is instead likely due to the pMHC reactivity skew in the altered TCR repertoire. In summary, our computational model and experimental data together suggest that the effect of TdT on chronic infection control is pathogen specific and that this may ultimately be a function of whether or not the pathogen can be cleared by responding T cells late in infection.

## Discussion

N-nucleotides added by TdT during V(D)J gene segment recombination contribute enormously to the diversification of the TCR repertoire [11]. Yet, despite the fact that TdT is found in all jawed vertebrates with adaptive immune systems studied thus far [12], the specific contexts in which these non-germline TCRs are better poised to control pathogen replication have not been clear [10,16]. Here, we combined computational modeling and experimental approaches to investigate the temporal evolution of pMHC reactivities of responding T cells during infection and its impact on pathogen clearance. We developed a computational model that (1) produced time courses characteristic of infections with both acute and chronic pathogens and (2) incorporated a continuum-affinity formalism to track T cell pMHC-reactivity distributions over time. Using the *in silico* model we developed, we made 2 predictions that we tested experimentally. First, we showed that, while in acute infection, T cells with high pMHC reactivity predominate [31,46,47], during chronic infection, T cells with low pMHC reactivity contribute disproportionately. Second, we found that the removal of low pMHC-reactivity T cells leads to a delay in chronic, but not acute, pathogen clearance in the model, which we replicated in infected mice when T cells were TdT deficient. Importantly, our data corroborate prior experimental work showing no differences in clearance by TdT KO mice of the acute viral pathogens vesicular stomatitis, Sendai, influenza A, and LCMV-WE [16,21]. Thus, while it has been proposed that a TdT-deficient TCR repertoire may have specific “holes” with regard to antigen specificities represented, this has so far not been supported by experimental evidence. Indeed, without accounting for possible differences in T cell clonotype precursor frequencies, our model predicts longer times to clearance for chronic pathogens. Our work therefore suggests a hitherto undescribed benefit for TCR repertoire diversification by TdT in chronic infection control. Importantly, our modeling and experimental data also suggest that TdT-dependent TCRs do not benefit host responses to chronic pathogens in the specific case where T cells exert limited control over pathogen replication in the chronic stages of infection.

Although our model was fit to viral replication data from acute and chronic strains of LCMV [22], some of its conclusions may be generalizable to other pathogens. For instance, we found that by retuning our model to reproduce *C*. *neoformans*, a pulmonary fungal pathogen, replication, we similarly observed a shift toward T cells with lower foreign pMHC reactivity in the chronic stages of infection. In line with this result, following infection with *C*. *neoformans*, CD4^+^ T cells with low self-pMHC reactivity, and thus presumably with lower foreign reactivity [34], predominated among the responding effector T cells during the chronic infection phase. These experimental findings expand on previous work suggesting that, during chronic infection, T cells with lower pMHC-reactivity predominate in both CD4^+^ [37] and CD8^+^ T cells [38,39].

Notably, in our current model, we did not distinguish between CD4^+^ and CD8^+^ T cell responses, and it is possible that there are key differences between these T cell subsets with regard to the role of TdT and functional biases among TdT-generated TCRs, which need to be further explored. For instance, we found evidence that CD4^+^ TdT KO T cells may be more prone to functional exhaustion upon chronic antigen stimulation given greater PD1 expression on activated CD4^+^ T cells, although no difference in PD1 expression was detected for activated CD8^+^ T cells. It should be noted, however, that while PD-1 expression levels can serve as a gauge of T cell exhaustion, it is increasingly appreciated that surface marker expression alone is insufficient to conclusively describe the state of T cell exhaustion [48–50]. A more comprehensive analysis for phenotyping of these TdT KO T cells (for instance, by assessing their transcriptional and epigenetic states during chronic infection) will be useful to further investigate the susceptibility of these cells to exhaustion relative to WT T cells as our computational modeling and experiments suggest.

It is also worth noting that our model did not consider memory T cells following pathogen clearance, and experimental data suggest that memory T cells are differentially selected for in terms of their reactivity to pMHC, resulting in a final steady-state pMHC-reactivity distribution of memory T cells that differs from the starting naïve T cell pMHC reactivity distribution [4,31,34]. Of note, while our model does not include all aspects of the host–pathogen response, such as the effects of host age-related changes in T cell precursor sizes and responses, phenotypic complexities of different stages of T cell exhaustion, antigen abundance and immunodominance hierarchies, and pathogen evolution to evade host immunity [51–53], the model could be extended by incorporating some of these additional phenomena in order to study their individual contributions to the control of acute or chronic pathogens in the context of a pMHC-reactivity continuum. Additionally, the effect of TdT deficiency in the B cell receptor repertoire and its impact on acute or chronic pathogen clearance would be interesting to investigate in future studies. Further, while the difference in host survival observed between WT and TdT KO mice was likely due to alterations in the pMHC reactivity profile of the responding T cell population as predicted by the computational model, whether parallel changes to the B cell receptor repertoire contribute to this difference remains to be tested. Moreover, although our LCMV-Cl13 viral load data qualitatively matched predicted pathogen loads from our model and provided support for delayed pathogen clearance in the absence of TdT, a more direct test of this effect would involve determining clearance time points in mice with either WT or TdT KO T cells, although these experiments would be particularly time- and resource-intensive with currently available techniques. Finally, while our simulations and experimental data propose that modulation of foreign antigen reactivity is one benefit of TdT-mediated TCR repertoire diversification, we could not rule out the existence of other affinity-independent advantages of TdT. For instance, whether TdT plugs “holes” in the TCR repertoire that would otherwise be exploited by as-yet untested pathogens remains an intriguing and open question.

A TdT-deficient repertoire and its consequences for pathogen control are relevant not only for understanding the broad evolutionary conservation of TdT across vertebrates but also in the context of neonatal immunity, given that the TCR repertoire is initially generated in the absence of TdT. TdT expression is first detected in thymocytes in mice and humans 3 to 5 days after birth and after 20 weeks of gestation, respectively [54,55]. Lacking N-nucleotide additions, neonatal TCR sequences are shorter, are more likely to be shared between individuals (public) [56,57], and are more cross-reactive [58]. Interestingly, in line with TdT KO T cells having greater pMHC reactivity, it has been shown that the neonatal repertoire is more self-reactive due to a greater affinity for pMHC, and neonatal T cells more prone to tolerance [59]. To what extent the neonatal TCR repertoire versus other epigenetic or transcriptional differences described in neonatal compared to adult T cells play a role in altered responses to infection requires further analysis.

Our work revealed the effects of varying features of the pMHC-reactivity distribution of responding T cells on pathogen clearance and suggested a differential role between T cells with low- and high-reactivity to pMHC during different phases of the immune response. This is particularly intriguing, given recent observations that in both mice and humans, both CD4^+^ and CD8^+^ T cells with lower self-pMHC reactivity (low CD5 surface levels) express higher levels of *Dntt*, the gene encoding TdT [36,60,61]. Thus, differences in TdT expression level during development in individual thymocytes may ultimately contribute to the numbers of N-nucleotides inserted into the recombining TCR and be a critical variable impacting strength of pMHC reactivity. To provide additional insight into whether there are indeed different roles for TdT-mediated versus germline-encoded TCRs during infection, comprehensive TCR sequencing studies will be a powerful tool. Indeed, recent work using machine learning has shown that CD4^+^ T cells with higher self-pMHC-reactivity have fewer N-nucleotide additions on average when compared to T cells with lower reactivity to pMHC and that longer TCR sequences with a greater number of N-nucleotide additions predominate in chronic infection [62]. Overall, our model formalism provides a foundation for further studies of T cell pMHC-reactivity distributions over the course of an immune response, and it will be particularly interesting to investigate whether, as our model suggests, TdT-dependent TCRs are important in the control of other chronic pathogens and are perhaps making underappreciated contributions in settings such as cancer and autoimmunity.

## Materials and methods

### Mice

C57BL/6, CD45.1^+^, Thy1.1^+^, and TCRβ KO mice [63] were purchased from Jackson Laboratories (Bar Harbor, ME). The TdT KO mice were shared by Dr. A. Feeney (The Scripps Research Institute) [64], and the JH KO mice were shared by Dr. J. Fritz (McGill) [65]. All mice were on a C57BL/6 background, bred in-house, and experiments performed at 6 to 12 weeks of age with both males and females. Animal housing, care, and research were in accordance with the Guide for the Care and Use of Laboratory Animals, and all procedures performed were approved by the McGill University Animal Care Committee (Animal Use Protocol number #MCGL-7570). All procedures conformed to the regulatory standards set by the Canadian Council on Animal Care.

### Pathogen stocks and infections

#### LCMV

LCMV-Arm and LCMV-Cl13 strains were propagated from stocks provided by Dr. M. Richer (University of Indiana) on BHK-21 or L929 cells (ATCC). Briefly, virus was added at MOI 0.01, incubated for 90 minutes in serum-free media at 37°C in 5% CO_2_, then topped up with complete media for incubation for another 48 hours before harvesting the supernatant. BHK-21 cells were cultured in EMEM supplemented with 0.1% penicillin/streptomycin, 1% L-glutamine, 1% nonessential amino acids, 1% sodium pyruvate, and 10% FBS and maintained at 37°C in 5% CO_2_. L929 cells were cultured in RPMI supplemented with 10% FBS, 1% L-glutamine, and 1% penicillin/streptomycin. Mice were infected with 2 × 10^5^ plaque-forming units (PFUs) of LCMV-Arm by intraperitoneal injection or 2 × 10^6^ PFU by intravenous injection for LCMV-Cl13 as previously described [22,66]. Mice were bled by either tail artery or cardiac puncture into sterile Eppendorf tubes kept on ice, blood was spun down at 12,000 rpm for 10 minutes, and serum aliquoted and frozen for viral titer determination. Spleens were collected in 1% RPMI and weighed. Spleens were placed in Lysing Matrix D tubes (MP Biomedicals) and homogenized with a MagNA Lyser (Roche) at 6,000 rpm for 40 seconds. Spleen homogenate was then spun down at 12,000 rpm for 10 minutes at 4°C, and supernatant was transferred to a separate sterile tube and respun at 12,000 rpm for 10 minutes at 4°C, then aliquoted and frozen for viral titer determination. Viral titers (stocks used, mouse serum, and tissue samples) were determined by plaque assay with Vero cells [26]. Briefly, Vero cell monolayers were infected with 100 μL of serially diluted serum (1 in 10 dilutions from 10^−1^ to 10^−7^) and incubated for 90 minutes at 37°C in 5% CO_2_. Infected cells were then overlaid with 1% agarose (Wisent) and incubated for 3 days at 37°C in 5% CO_2_. A second agarose overlay supplemented with 1% neutral red was then added and cells incubated for 24 hours at 37°C in 5% CO_2_, after which plaques were counted.

#### C. neoformans

The H99 strain was provided by K. Kwon-Chung (NIH). Frozen stocks (−80°C) were prepared in 15% glycerol from fresh cultures from a YPD agar plate. Three days before infection, *C*. *neoformans* was scraped from the frozen stock and streaked onto a YPD agar plate. One day prior to infection, a single colony was inoculated and incubated for 12 to 16 hours at 30°C with continuous agitation in YPD broth. Immediately before infection, *C*. *neoformans* was resuspended in cold PBS. Mice were then anesthetized with isoflurane and infected by intrapharyngeal aspiration with 5 × 10^3^ for all experiments except the survival experiment where mice were infected with 5 × 10^4^ colony-forming units (CFUs) in 20 μL of PBS. Mice were euthanized and tissue collected 20 days after infection. For the survival experiment (**Fig 5D**), mice were monitored every 1 to 2 days for 50 days and killed when their weight loss exceeded 20% of their initial body weight or when they exhibited signs of irreversible disease. *C*. *neoformans* CFUs in harvested tissues were determined as previously described [30].

### Lymphocyte isolation

For the *C*. *neoformans* infections, prior to harvest, an intravascular stain using 2.5 μg anti-CD45 (30F11) was performed as previously described [67]. Infected lungs were harvested in cold PBS and minced with scissors. Lung was then digested at 37°C with agitation for 30 minutes in digestion buffer (1 mg/mL collagenase D, 50 U/mL DNase I, 1 mg/mL hyaluronidase, 1% L-glutamine, 1% pen/strep in RPMI). Tissue was then passed through a 100-μm filter with PBS supplemented with 1% FBS and resuspended in 10 mL of 37% Percoll in RPMI. Samples were centrifuged at 3,000 rpm for 20 minutes at 22°C. ACK lysis buffer (Life Technologies) was added for 3 minutes, samples washed with PBS, refiltered, and resuspended in complete RPMI (10% FBS, 1% L-glutamine, 1% HEPES buffer, 1% pen/strep, 1% sodium pyruvate, 1% nonessential amino acids, 0.1% 2-mercapto-ethanol 1000X solution). Dilution of single-cell suspensions at 1:10 in Trypan Blue and manual counting of live cells (Trypan Blue-negative) on a hemacytometer was used to determine total cell counts.

For the LCMV infections, spleen and peripheral lymph nodes (inguinal, axillary, brachial, and mesenteric) were collected and passed through a 70-μm filter with 1% RPMI (1% penicillin/streptomycin, 1% L-glutamine, and 1% FBS). ACK lysis buffer (Life Technologies) was added for 3 minutes, samples washed with PBS, refiltered, and resuspended in 1% RPMI. Dilution of single-cell suspensions at 1:10 in Trypan Blue and manual counting of live cells (Trypan Blue-negative) on a hemacytometer was used to determine total cell counts.

### Bone marrow chimeras

BM was collected from the femurs and tibias of donor mice (either JH TdT double KO, TCRβ KO, or B6 WT) by flushing the marrow from the bones with cold 1% RPMI. BM cells were then passed through a 70-μm filter with 1% RPMI and cell counts determined as above. Recipient mice (either B6 CD45.1^+^, B6 Thy1.1^+^, or TCRβ KO CD45.1^+^) were irradiated twice at 550 rads 3 hours apart and reconstituted with a 1:1 mix of 2.5 × 10^6^ cells per genotype that were injected intravenously within 5 hours of the first irradiation. To establish the WT chimera (WT T cells and B cells), B6 and TCRβ KO BM cells were mixed at equal proportions; to make the T cell restricted TdT KO chimeras (WT B cells), BM cells from JH TdT double KO mice were mixed 1:1 with TCRβ KO BM cells (Fig 4F). To generate the competitive development chimeras (**S4A Fig**), B6 CD45.1^+^ and TdT KO CD45.2^+^ BM cells were mixed in equal proportions and injected into Thy1.1^+^ recipient mice. Recipient mice were given neomycin water (2 g/L) 2 days prior to BM transfer and kept on the antibiotic water for 2 weeks following transfer. Mice were used 6 weeks (for thymocyte experiments) or 8 to 12 weeks (for infection experiments) post irradiation and BM reconstitution.

### Flow cytometry

Samples were incubated in Fixable Viability Dye (eFluor 780, Life Technologies) diluted in PBS for 20 minutes at 4°C. Extracellular antibodies were diluted in FACS buffer (2% FBS and 5 mM EDTA in PBS) with Fc Block (Life Technologies) and incubated for 30 minutes at 4°C. For intracellular staining, samples were then incubated in FoxP3 Transcription Factor Fixation/Permeabilization Concentrate and Diluent (Life Technologies) for 30 minutes at 4°C. Intracellular antibodies were diluted in Permeabilization Wash Buffer (Life Technologies), and samples were incubated for 30 to 60 minutes at 4°C. Directly conjugated antibodies used were as follows: TCRb (H57-597), CD4 (RM4.5), CD8a (53–6.7), CD5 (53–7.3), Foxp3 (FJK-16s), CD44 (IM7), CD62L (MEL-14), CD25 (PC61.5), CD45.1 (A20), CD45.2 (104), PD-1 (29F.1A12), B220 (RA3-6B2), NK1.1 (PK126), CD69 (H1.2F3), CD3 (145-2C11), Thy1.1 (OX-7), Thy1.2 (30-H12). Monomers of MHC class I H-2D^b^ GP33 and MHC class II I-A^b^ GP66 were obtained from the National Institutes of Health Tetramer Core, and tetramers were generated using streptavidin-APC (1 mg/mL, Invitrogen). For tetramer staining, samples were incubated for 15 minutes at 37°C or 1 hour at room temperature in PBS supplemented with 2% FBS for GP33:D^b^ or GP66:I-A^b^, respectively, prior to viability staining. For all flow cytometry experiments, cells were acquired using an LSRFortessa (BD Biosciences) or an Aurora (Cytek Biosciences) and analyzed with FlowJo software (BD Biosciences). Compensation was performed in FACSDiva Software (BD Biosciences) or in SpectroFlo (Cytek Biosciences). Manual compensation correction was performed in the FlowJo software. Relative fluorescence intensity was determined by normalizing expression levels in comparison with specific control population (defined in the legend where applicable).

### Cell sorts

Cell sorts were performed as previously described [36]. Briefly, lymphocytes from Thy1.1^+^ (for *C*. *neoformans* infection) or CD45.1^+^ (for *C*. *neoformans* and LCMV-Cl13 infections) congenic mice were isolated in single-cell suspension as described. Spleens and lymph nodes (inguinal, axillary, brachial, mesenteric, and cervical) were pooled from 15 (for *C*. *neoformans* infection) or 37 (for LCMV-Cl13 infection) mice for each congenic marker. Cells were then magnetically enriched for total CD4^+^ T cells or total T cells (Stemcell EasySep CD4^+^ T cell Enrichment kit, Miltenyi Biotec CD4^+^ T cell Isolation Kit, or Stemcell EasySep T cell Enrichment kit). Enriched T cells were stained with surface antibodies for 1 hour at 4°C. Naïve CD4^+^ T cells were sorted on singlets, CD4^+^, CD8^−^, CD25^−^, CD62L^hi^, CD44^lo^, and 20% CD5^lo^ or CD5^hi^. Sorts were performed on a FACS Aria III (BD Bioscience). All cell populations were sorted to >90% purity.

### Adoptive cell transfers

#### *C*. *neoformans* infection

All donors and recipients were sex matched. A total of 15 CD45.1^+^ or Thy1.1^+^ mice were used as donors to obtain a total of 8 to 14 × 10^6^ cells for each of 20% CD5^lo^ and 20% CD5^hi^ cells sorted as described above. Sorted CD5^lo^ and CD5^hi^ CD4^+^ T cells were then mixed in a 1:1 ratio. A total of 4 to 7 × 10^6^ cells of each sorted population was adoptively transferred into CD45.2^+^ Thy1.2^+^ recipients that were infected with 5 × 10^3^ CFU of *C*. *neoformans* 3 days prior to transfer. Cells were isolated from the lungs of recipient mice 20 days post-infection.

#### LCMV-Cl13 infection

All donors and recipients were sex matched. A total of 37 CD45.1^+^ mice were used as donors to obtain a total of 14.3 to 18.7 × 10^6^ cells for each of 20% CD5^lo^ and 20% CD5^hi^ CD4^+^ T cells sorted as described above. A total of 3.5 to 4.7 × 10^6^ cells were adoptively transferred into CD45.2^+^ recipients. Mice were infected with 2 × 10^6^ PFU of LCMV-Cl13 1 day after cell transfer. Cells were isolated from the spleens of recipient mice 59 days post-transfer.

### Statistical analyses of experimental data

Group comparisons were performed using Prism V9 (GraphPad). The cutoff for significance considered was *P* < 0.05. Information about implemented statistical tests and sample sizes for individual experiments is provided in the figure legends.

### Computational modeling

To study a continuum of antigen-specific T cell affinities in the context of acute versus chronic pathogen infections, we used the following system of integro-differential equations based on Fig 1:

$$\frac{dP}{dt}=r_{P}P\left(1-\frac{P}{P_{max}}\right)-\kappa_{P}\int_{k_{min}}^{k_{max}}E(t,k)\frac{P}{P+ak}dk$$

$$\frac{\partial E(t,k)}{\partial t}=\sigma_{E}(k)+r_{E}E(t,k)\frac{P}{P+k}-\delta_{E}E(t,k)$$ (1)

$$-\kappa_{E}(k)E(t,k)\frac{P}{P+bk}-\varepsilon E(t,k)\int_{k_{min}}^{k_{max}}E(t,k)dk.$$ (2)

This was implemented in a manner similar to the models presented in [68,69]. In Eqs (1) and (2), *P*(*t*) represents the pathogen load in time, and *E*(*t*, *k*) represents the time-dependent reactivity-continuum of effector T cells, with T cell pMHC reactivity taken to be proportional to the quantity 1/*k*, where *k* is the pathogen load for half-maximum activation of T cells. To facilitate understanding of pMHC-reactivity distributions, we defined a new, unitless quantity *a*_*k*_ (whose magnitude is equivalent to 1/*k*) as a measure for T cell reactivity to pMHC [29]. While this quantity is related to the overall binding avidity between T cells and APCs, we considered only affinity-related changes in TCR binding strength and thus used the term pMHC reactivity to avoid confusion with other factors that can affect binding avidity. For simplicity, in Eq (1), we assumed a logistic growth of the pathogen (with replication rate *r*_*P*_ and carrying capacity *P*_max_) as was done in previous models of LCMV infection [70,71]. Clearance of the pathogen by T cells was described by a product of the T cell number (*E*) and an increasing first-order Hill function of pathogen load with a maximum rate *κ*_*P*_, and a half-maximum pathogen load *a*∙*k* (where *a* is a scaling factor). We impose the condition that the pathogen replication rate (*r*_*P*_) for chronic pathogens to be larger than that for acute pathogens, as suggested previously in the case of LCMV-Cl13 versus LCMV-Arm, respectively [25,72].

The terms included in effector cell dynamics of Eq (2) were pMHC reactivity-dependent thymic input (*σ*_*E*_(*k*), i.e., the source of antigen-specific naïve T cell precursors arising from the thymus), T cell replication that depends on pathogen load according to a first-order Hill function (with maximum replication rate *r*_*E*_ and half-maximum activation *k*), natural turnover (with a rate *δ*_*E*_), and an intercellular competition term (with a rate *ε*). The parameter *κ*_*E*_ denotes the rate at which the pathogen causes reduction in the number of active effector T cells able to provide antimicrobial immunity, either via T cell exhaustion, activation-induced cell death, or regulatory T cell intervention. The term *b*∙*k* represents the pathogen load at half the maximum inactivation rate of effector T cells, where *b*>1 is a proportionality constant.

To study the effects of T cell reactivity to pMHC in the context of acute versus chronic infection, we denoted the spectrum of effector T cells in time by *E*(*t*, *k*), where *k* = 1/*a*_*k*_ is assumed to be proportional to the reciprocal of T cell reactivity to pMHC consistent with [29], *k*_max_ is the maximum value of *k* (corresponding to T cells with the lowest reactivity to pMHC) and *k*_min_ is the minimum value of *k* (corresponding to T cells with highest reactivity to pMHC). Eqs (1) and (2) are simulated by discretizing the allowable values of pMHC reactivity within the range defined by 1/*k*_max_ and 1/*k*_min_, wherein the solution is obtained by integrating a high-dimensional system of ordinary differential equations (see **S1 Text**).

### Model parameters and numerical implementation

The 2 pMHC reactivity–dependent parameters, *σ*_*E*_ and *κ*_*E*_, were defined to be functions of T cell reactivity to pMHC (*a*_*k*_) (**Fig 1B and 1C**). A bell-shaped function of pMHC reactivity that mimics a log-normal distribution was used to assign values for thymic input *σ*_*E*_, while T cell exhaustion *κ*_*E*_ was first sampled from an exponential distribution and then sorted in an ascending order (such that higher-affinity T cells are more susceptible to losing their effector functions than their lower-affinity counterparts). This latter claim is supported by the fact that activation-induced cell death [23] and T cell exhaustion [22,24] scale with the strength of antigenic stimulation. The use of a uniform distribution for *κ*_*E*_ produced very similar results (**S6 Fig**). S1 Table summarizes the meanings of the different parameters and their values used to generate model results. Model parameters were obtained by genetic algorithm fitting to LCMV-Arm and LCMV-Cl13 serum data adapted from [22] (**S1A and S1B Fig**). To simulate the ΔTCR repertoire shown in Fig 4C, the thymus input parameter *σ*_*E*_ was modulated by an arctan function such that

$$\sigma_{E}^{\Delta TCR}(k)={\frac{1}{2}\sigma }_{E}(k)\left[1-\arctan\left(\frac{\log_{10}k-\log_{10}k_{mode}}{0.25}\right)\right],$$

where *k*_mode_ is the value of the parameter *k* at which *σ*_*E*_(*k*) peaks, determined by parameter fitting (for more details, see **S1 Text**). The new parameter $\sigma_{E}^{\Delta TCR}(k)$ is subsequently renormalized such that the total thymic input across all T cells remains constant, i.e.,

$$\sigma_{E}^{\Delta TCR}(k)\to \sigma_{E}^{\Delta TCR}(k)\left(\int_{k_{min}}^{k_{max}}\sigma_{E}(k)dk\right)/\left(\int_{k_{min}}^{k_{max}}\sigma_{E}^{\Delta TCR}(k)dk\right).$$

### Software and code availability

MATLAB was used to simulate the model equations and perform numerical analyses. Steady-state and bifurcation analyses were carried out using XPP/AUTO, a freeware available at http://sites.pitt.edu/~phase/bard/bardware/xpp/xpp.html). Genetic algorithm fitting was performed using Compute Canada’s Cedar cluster. MATLAB codes used to produce violin plots are available at http://github.com/bastibe/Violinplot-Matlab. All results and figures generated by the computational model, including parameter fitting, time series simulations, and model analyses, can be exactly reproduced using the source code deposited to Zenodo and available at https://doi.org/10.5281/zenodo.10202100.

## Supporting information

S1 Fig Model fitting, adoptive cell transfer experiment setup details, and CD5hi/lo sorted CD4 T cell contribution during chronic LCMV-Cl13 infection. (**A, B**) Serum viral load data in mice infected with LCMV-Arm (**A**) or LCMV-Cl13 (**B**) digitized from [22], shown as open circles, overlayed on the time series simulations of Eqs (1) and (2) presented in the main text. These simulations were generated using parameter values (S1 Table) obtained from fitting the model to digitized data shown in (**A**) and (**B**) with the implementation of the genetic algorithm (see Model parameters and fitting in **S1 Text** for details). Note that the difference between the 2 curves was generated by altering the pathogen replication rate parameter and initial pathogen load. (**C**) Median time to clearance of 100 simulations for acute and chronic infections (error bars = 95% confidence intervals). (**D**) Flow cytometry panels showing sort purities of transferred CD45.1^+^ or Thy1.1^+^ CD5^lo^ and CD5^hi^ naïve CD44^lo^ CD62L^+^ CD4^+^ T cells into *C*. *neoformans*-infected mice. (**E**) Representative flow cytometry plot of CD5^lo^ CD45.1^+^ and CD5^hi^ Thy1.1^+^ transferred T cell populations preinjection mix into recipient *C*. *neoformans* infected mice (**F**) *C*. *neoformans* pathogen loads in the lungs of infected mice at experimental endpoint (20 days post-infection), from 2 independent experiments (*n* = 8 mice). (**G**) Schematic of experimental approach for adoptive cell transfer of sorted CD5^lo^ and CD5^hi^ naïve CD4^+^ T cells (CD45.1^+^) into congenic CD45.2^+^ recipient mice. Mice were infected with LCMV-Cl13 1 day post-transfer. (**H**) Representative flow cytometry plot of activated (CD44^hi^) CD5^lo^ and CD5^hi^ transferred CD4^+^ T cell populations 59 days post-transfer with CD5 expression levels shown as a histogram and mean fluorescent intensities (MFI) indicated in blue and red text. (**I**) Ratio of activated (CD44^hi^) CD5^lo^ or CD5^hi^ transferred T cells relative to naïve (CD62L^hi^ CD44^lo^) CD5^lo^ or CD5^hi^ transferred T cells 59 days post-transfer. *P* = 0.11 computed using a Mann–Whitney test, *n* = 4 mice. The experimental data underlying this figure can be found in S1 Data. See Methods to access code used to produce model simulations in this figure.
(PDF)

S2 Fig Bifurcation analysis of the one-clone system. (**A**) Pathogen levels at steady state as a function of pMHC reactivity (*a*_*k*_ = 1/*k*); solid black lines represent branches of attracting (stable) equilibria, while dashed lines represent branches of repelling (unstable) equilibria. The upper and lower levels of pathogen load can coexist (in the form of bistability) in the upper range of pMHC reactivity, which one of these 2 steady states can be attained depend on the initial conditions of pathogen load and T cell count. (**B**) Pathogen levels at steady state as a function of the pathogen-dependent effector T cell depletion, *κ*_*E*_, when *a*_*k*_ = 10^−2.98^; as before, solid black lines represent branches of attracting (stable) equilibria, while dashed lines represent branches of repelling (unstable) equilibria. (**C**) Two-parameter bifurcation of steady-state level of pathogen load with respect to the depletion rate *κ*_*E*_ and pMHC reactivity parameter, *a*_*k*_. Gray-shaded region represents the regime of coexistence between the upper and lower levels of pathogen load (i.e., the bistable regime) seen in (**A**) and (**B**). Overlayed is the trajectory of the average pMHC reactivity and average depletion rate of the ensemble of T cells of the full system, starting from the filled black circle (arrows indicate direction of motion). See Methods to access code used to produce model simulations in this figure.
(PDF)

S3 Fig Effects of removing T cells with low pMHC-reactivity. (**A**) Distributions of initial T cell count prior to infection as a function of pMHC reactivity obtained by successively removing low-reactivity T cells using different cutoff thresholds from the model’s preinfection repertoire and by reducing the pathogen replication rate *r*_*P*_ to 1.16 day^−1^ from its default value. Note that, since reducing *r*_*P*_ does not affect the initial T cell count, these distributions are identical to those shown in Fig 4A. (**B**) Time to pathogen clearance as a function of the cutoff threshold for T cell reactivity shown in (**A**). For each cutoff threshold, 50 simulation trials were performed as described in Fig 4. Notice the prominence of the acute cluster at high cutoff thresholds owing to a greater number of T cells with high pMHC reactivity; interestingly, this feature is not present in Fig 4B. **(C)** Distribution of T cell count prior to infection and with *r*_*P*_ reduced to 1.16 day^−1^, without keeping the total number of T cells conserved when removing the low-reactivity T cells at different cutoff thresholds. **(D)** Time to clearance as a function of the cutoff threshold of the pMHC reactivity shown in (**C**). Notice the disappearance of the acute cluster seen in (**B**) at high cutoff threshold values. See Methods to access code used to produce model simulations in this figure.
(PDF)

S4 Fig TdT-deficient T cells undergo more efficient positive selection. (**A**) Mixed BM chimeras possessing WT and TdT KO cells (reconstitution of irradiated mice with 1:1 ratio of BM cells) were generated, and the thymus was harvested 6 weeks after irradiation for each mouse. (**B, C**) RFI of surface CD5 expression on pre-selection DP thymocytes (left) and CD4^+^ and CD8^+^ SP thymocytes (right), relative to CD5 expression on WT double-negative thymocytes; lines connect WT and TdT KO cells from the same chimeric mouse (**B**). Ratio of TdT KO to WT pre-selection DP, CD4^+^ SP, and CD8^+^ SP thymocytes (**C**). Data are summarized from 2 independent experiments, *n* = 17. (**D**) Representative flow cytometry plots showing the percent of BM cells from each set of donor mice, namely, CD45.1^+^ cells from TCRβ KO mice and CD45.2^+^ cells from either WT or JH × TdT double KO mice. (**E**) Summary MFI of PD1 expression on WT and TdT KO tetramer-positive activated CD4^+^ (CD44^hi^ GP66:I-A^b+^) and CD8^+^ (CD44^hi^ GP33:D^b+^) T cells, *n* = 4–5 chimeras. (**F**) RFI of surface PD1 expression on total activated (CD44^hi^) CD4^+^ and CD8^+^ T cells normalized to expression on activated (CD44^hi^) WT CD4^+^ or CD8^+^ T cells, respectively. Data are summarized from 4 independent experiments. *n* = 14 chimeras. (**G**) Pathogen loads generated from 50 model simulations at time points equivalent to those indicated in Fig 4J, using either the WT or the ΔTCR repertoire configurations (Fig 4C). Horizontal lines indicate mean values. (**H**) Modified experimental approach from Fig 4F by using TCRβ KO mice as irradiated BM recipients. (**I**) LCMV-Cl13 viral loads in the serum of recipient TCRβ KO mice reconstituted with WT or TdT KO T cells at day 41 post-infection. *P* values indicated were computed using Wilcoxon matched-pairs signed rank test (**B**), Friedman test (**C**), Mann–Whitney test (**E** and **F**), and two-tailed Wilcoxon rank sum test on geometric means (**H**). ns = not significant, * *P* < 0.05, *** *P* < 0.001. The experimental data underlying this figure can be found in S1 Data. See Methods to access code used to produce model simulations in this figure. BM, bone marrow; DP, double-positive; KO, knockout; LCMV, lymphocytic choriomeningitis virus; MFI, mean fluorescent intensity; RFI, relative fluorescence intensity; SP, single-positive; TdT, terminal deoxynucleotidyl transferase; WT, wild type.
(PDF)

S5 Fig Alternative changes to the TCR repertoire in the model produce outcomes that do not match the data in mice with TdT-deficient T cells. (**A, D**) Altered TCR repertoire obtained by either reducing the number of clonotypes by decreasing *N* to 50, resulting in fewer clones across the entire pMHC reactivity range while keeping the total T cell count constant (**A**), or reducing precursor frequency across all pMHC reactivity values by decreasing *σ*_*E*,tot_ 10-fold to 2.97 cells day^−1^ (**D**). (**B**, **E**) Model simulations comparing representative pathogen load traces of WT (gray) and ΔTCR repertoires (red) configurations in (**A**) and (**C**) during acute (top) or chronic (bottom) infection when the number of clones is reduced (**B**) or when precursor frequencies are reduced (**E**). Note the overlap of the 2 curves in (**B**). (**C**, **F**) Time to clearance of acute (left) or chronic (right) infections for 50 model simulations (log-values of initial pathogen loads randomized to ±10% of log-values in S1 Table) from WT and ΔTCR repertoire systems associated with reducing number of clones (**C**) or precursor frequencies (**F**). Horizontal lines indicate mean values. ns = not significant; ***, *P* < 0.001. *P* values computed using the Wilcoxon rank sum test. See Methods to access code used to produce model simulations in this figure. pMHC, peptide-major histocompatibility complex; TCR, T cell receptor; TdT, terminal deoxynucleotidyl transferase; WT, wild type.
(PDF)

S6 Fig Exhaustion rates sampled from a uniform distribution do not qualitatively alter model results. (**A**) Function depicting pMHC reactivity-dependent exhaustion rate, *κ*_*E*_, when sampling from a uniform distribution (with a lower bound of *κ*_*E*,min_ as in S1 Table, and upper bound *κ*_*E*,max_ set to 6.67 day^−1^) and sorting in ascending order. (**B**, **C**) Pathogen load (top) and pMHC-reactivity distribution (bottom) obtained by simulating the model response to acute (**B**) or chronic (**C**) pathogen, when *κ*_*E*_ was sampled from uniform distribution. Chronic replication rate (*r*_*P*_) was reduced to 1.15 day^−1^; all other parameters were kept at their default values shown in S1 Table. (**D**, **E**) Effect of varying the pMHC-reactivity mode (**D**), as in Fig 3A, or of removing T cells with low pMHC-reactivity (**E**), as in Fig 4B, when *κ*_*E*_ was sampled from uniform distribution. Note that all results are consistent with those obtained by sampling *κ*_*E*_ from an exponential distribution as shown in Fig 1C. See Methods to access code used to produce model simulations in this figure.
(PDF)

S1 Table Parameter values used in model simulations. Values were determined by fitting model simulations to serum data in acute vs. chronic LCMV infection [22] using a genetic algorithm.
(DOCX)

S1 Movie (**A-F**) Time series simulations of the model during acute (**A**-**C**) or chronic (**D**-**F**) infection, showing pathogen loads (**A**, **D**), heat maps representing the relative proportion of T cells across pMHC reactivities (**B**, **E**), and evolution of T cell proportions as a function of pMHC reactivity at each time point (**C**, **F**). Overlayed traces in (**B**) and (**E**) represent the mean reactivity value in time, weighted by the proportion of T cells of given reactivity values. Dotted black lines denote the time at which the pathogen is cleared. See Methods to access code used to produce model simulations in this movie.
(MP4)

S2 Movie (**A, B**) Nullclines of the reduced, 2D, one-clone model plotted in logarithmic (**A**) and linear (**B**) scales over time during chronic pathogen replication. Nullclines vary over time since the parameters for pMHC reactivity (*a*_*k*_) and exhaustion rate (*κ*_*E*_) of the one-clone model were set to their respective weighted average values across all T cells of the full model at each time point. Given that the relative proportions of T cells of different pMHC reactivity values vary through time in the full model, the weighted averages of these parameters also change over time. Black lines represent pathogen load (*P*) nullclines, while the gray line represents the effector T cell (*E*) nullcline. Superimposed in green is the pathogen load and the total T cell count (across all values of pMHC reactivity) simulated from the full model across time. Refer to Analysis of the reduced one-clone, 2D model for more details. See Methods to access code used to produce model simulations in this movie.
(MP4)

S1 Data Original data used to plot experimental results. Each sheet summarizes the data plotted in individual panels containing experimental results within the main and supporting figures.
(XLS)

S1 Text Supporting information regarding the computational model. Description of model parameters and fitting, detailed numerical implementation, stability analysis of the full model, and analysis of a reduced, one-clone model.
(DOCX)

## Abbreviations

BM: bone marrow
CFU: colony-forming unit
DP: double-positive
LCMV: lymphocytic choriomeningitis virus
KO: knockout
PFU: plaque-forming unit
pMHC: peptide-major histocompatibility complex
SP: single-positive
TCR: T cell receptor
TdT: terminal deoxynucleotidyl transferase
