# Supplementary figures and images for: Chronic infection control relies on T cells with lower foreign antigen binding strength generated by N-nucleotide diversity
Source: PLoS Biol. 2024 Feb 1;22(2):e3002465. doi: 10.1371/journal.pbio.3002465 (PMC10833529; doi:10.1371/journal.pbio.3002465)

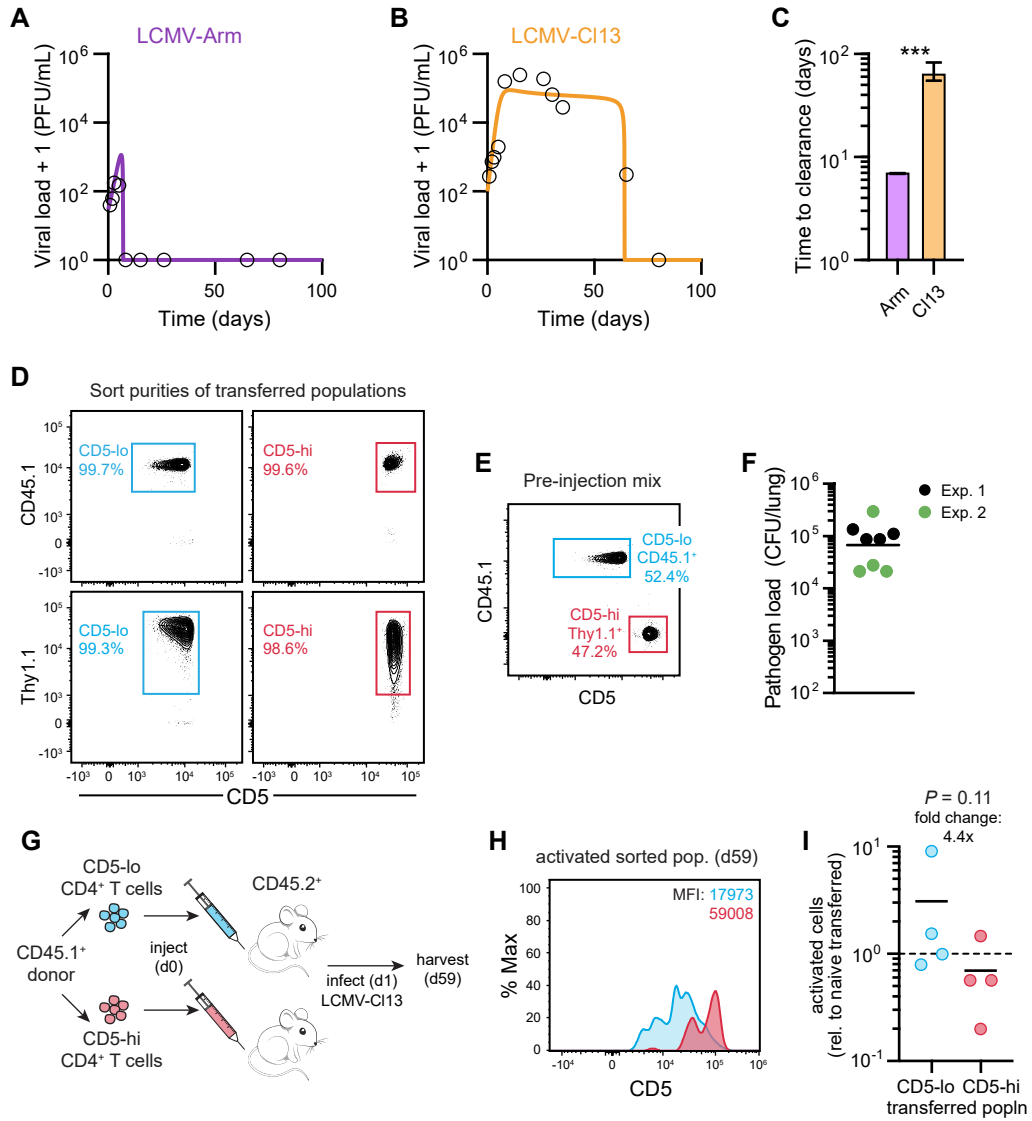

Supplement: S1 Fig — (A, B) Serum viral load data in mice infected with LCMV-Arm (A) or LCMV-Cl13 (B) digitized from [22], shown as open circles, overlayed on the time series simulations of Eqs (1) and (2) presented in the main text. These simulations were generated using parameter values (S1 Table) obtained from fitting the model to digitized data shown in (A) and (B) with the implementation of the genetic algorithm (see Model parameters and fitting in S1 Text for details). Note that the difference between the 2 curves was generated by altering the pathogen replication rate parameter and initial pathogen load. (C) Median time to clearance of 100 simulations for acute and chronic infections (error bars = 95% confidence intervals). (D) Flow cytometry panels showing sort purities of transferred CD45.1+ or Thy1.1+ CD5lo and CD5hi naïve CD44lo CD62L+ CD4+ T cells into C. neoformans-infected mice. (E) Representative flow cytometry plot of CD5lo CD45.1+ and CD5hi Thy1.1+ transferred T cell populations preinjection mix into recipient C. neoformans infected mice (F) C. neoformans pathogen loads in the lungs of infected mice at experimental endpoint (20 days post-infection), from 2 independent experiments (n = 8 mice). (G) Schematic of experimental approach for adoptive cell transfer of sorted CD5lo and CD5hi naïve CD4+ T cells (CD45.1+) into congenic CD45.2+ recipient mice. Mice were infected with LCMV-Cl13 1 day post-transfer. (H) Representative flow cytometry plot of activated (CD44hi) CD5lo and CD5hi transferred CD4+ T cell populations 59 days post-transfer with CD5 expression levels shown as a histogram and mean fluorescent intensities (MFI) indicated in blue and red text. (I) Ratio of activated (CD44hi) CD5lo or CD5hi transferred T cells relative to naïve (CD62Lhi CD44lo) CD5lo or CD5hi transferred T cells 59 days post-transfer. P = 0.11 computed using a Mann–Whitney test, n = 4 mice. The experimental data underlying this figure can be found in S1 Data. See Methods to access code used to p [file pbio.3002465.s001.pdf]

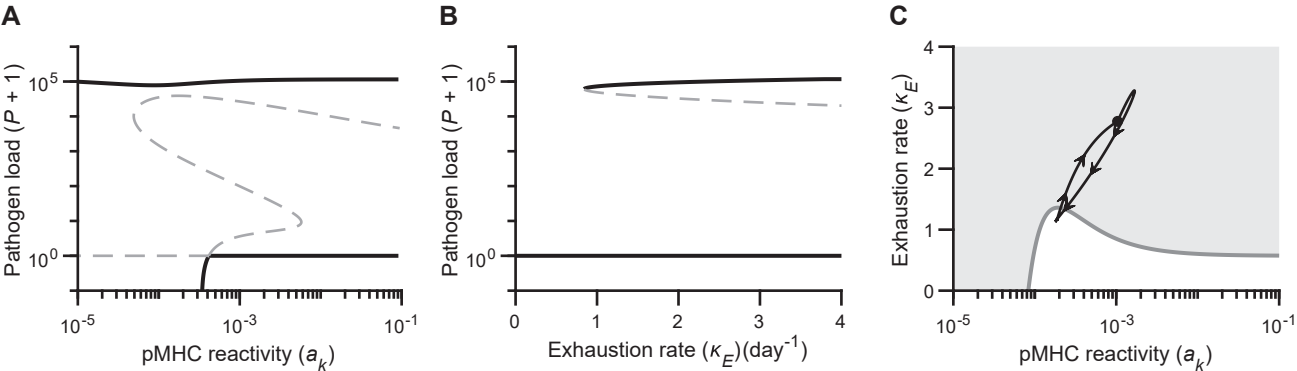

Supplement: S2 Fig — (A) Pathogen levels at steady state as a function of pMHC reactivity (ak = 1/k); solid black lines represent branches of attracting (stable) equilibria, while dashed lines represent branches of repelling (unstable) equilibria. The upper and lower levels of pathogen load can coexist (in the form of bistability) in the upper range of pMHC reactivity, which one of these 2 steady states can be attained depend on the initial conditions of pathogen load and T cell count. (B) Pathogen levels at steady state as a function of the pathogen-dependent effector T cell depletion, κE, when ak = 10−2.98; as before, solid black lines represent branches of attracting (stable) equilibria, while dashed lines represent branches of repelling (unstable) equilibria. (C) Two-parameter bifurcation of steady-state level of pathogen load with respect to the depletion rate κE and pMHC reactivity parameter, ak. Gray-shaded region represents the regime of coexistence between the upper and lower levels of pathogen load (i.e., the bistable regime) seen in (A) and (B). Overlayed is the trajectory of the average pMHC reactivity and average depletion rate of the ensemble of T cells of the full system, starting from the filled black circle (arrows indicate direction of motion). See Methods to access code used to produce model simulations in this figure. (PDF) [file pbio.3002465.s002.pdf]

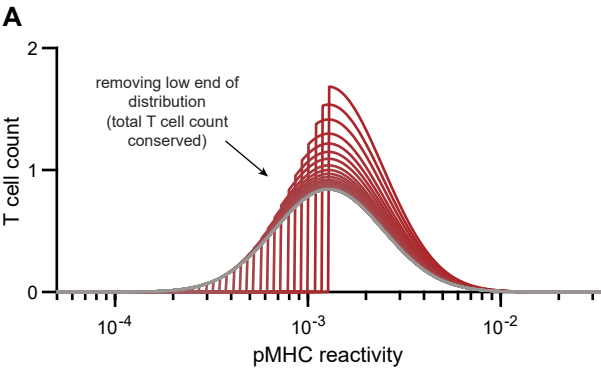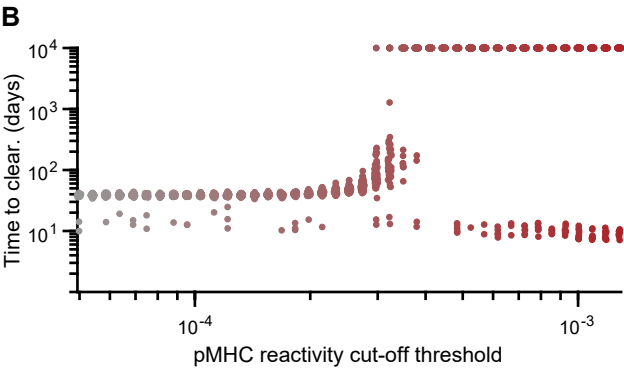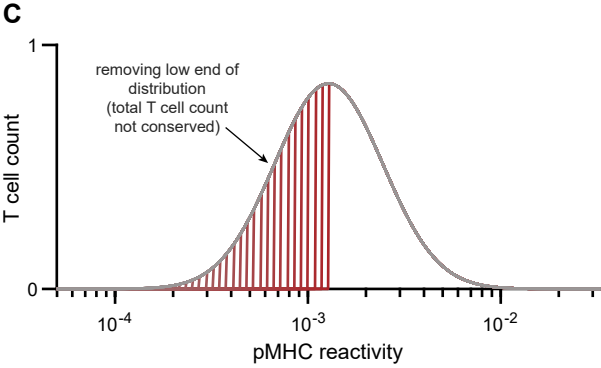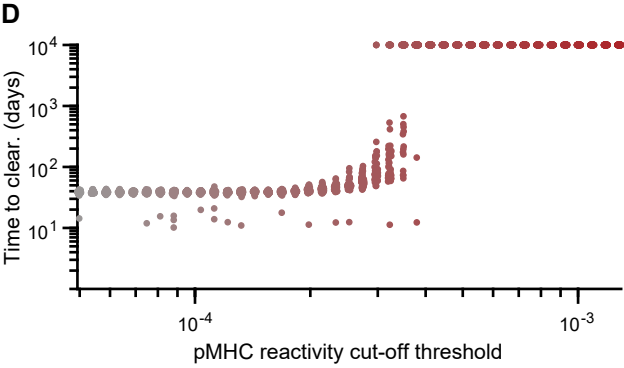

Supplement: S3 Fig — (A) Distributions of initial T cell count prior to infection as a function of pMHC reactivity obtained by successively removing low-reactivity T cells using different cutoff thresholds from the model’s preinfection repertoire and by reducing the pathogen replication rate rP to 1.16 day−1 from its default value. Note that, since reducing rP does not affect the initial T cell count, these distributions are identical to those shown in Fig 4A. (B) Time to pathogen clearance as a function of the cutoff threshold for T cell reactivity shown in (A). For each cutoff threshold, 50 simulation trials were performed as described in Fig 4. Notice the prominence of the acute cluster at high cutoff thresholds owing to a greater number of T cells with high pMHC reactivity; interestingly, this feature is not present in Fig 4B. (C) Distribution of T cell count prior to infection and with rP reduced to 1.16 day−1, without keeping the total number of T cells conserved when removing the low-reactivity T cells at different cutoff thresholds. (D) Time to clearance as a function of the cutoff threshold of the pMHC reactivity shown in (C). Notice the disappearance of the acute cluster seen in (B) at high cutoff threshold values. See Methods to access code used to produce model simulations in this figure. (PDF) [file pbio.3002465.s003.pdf]

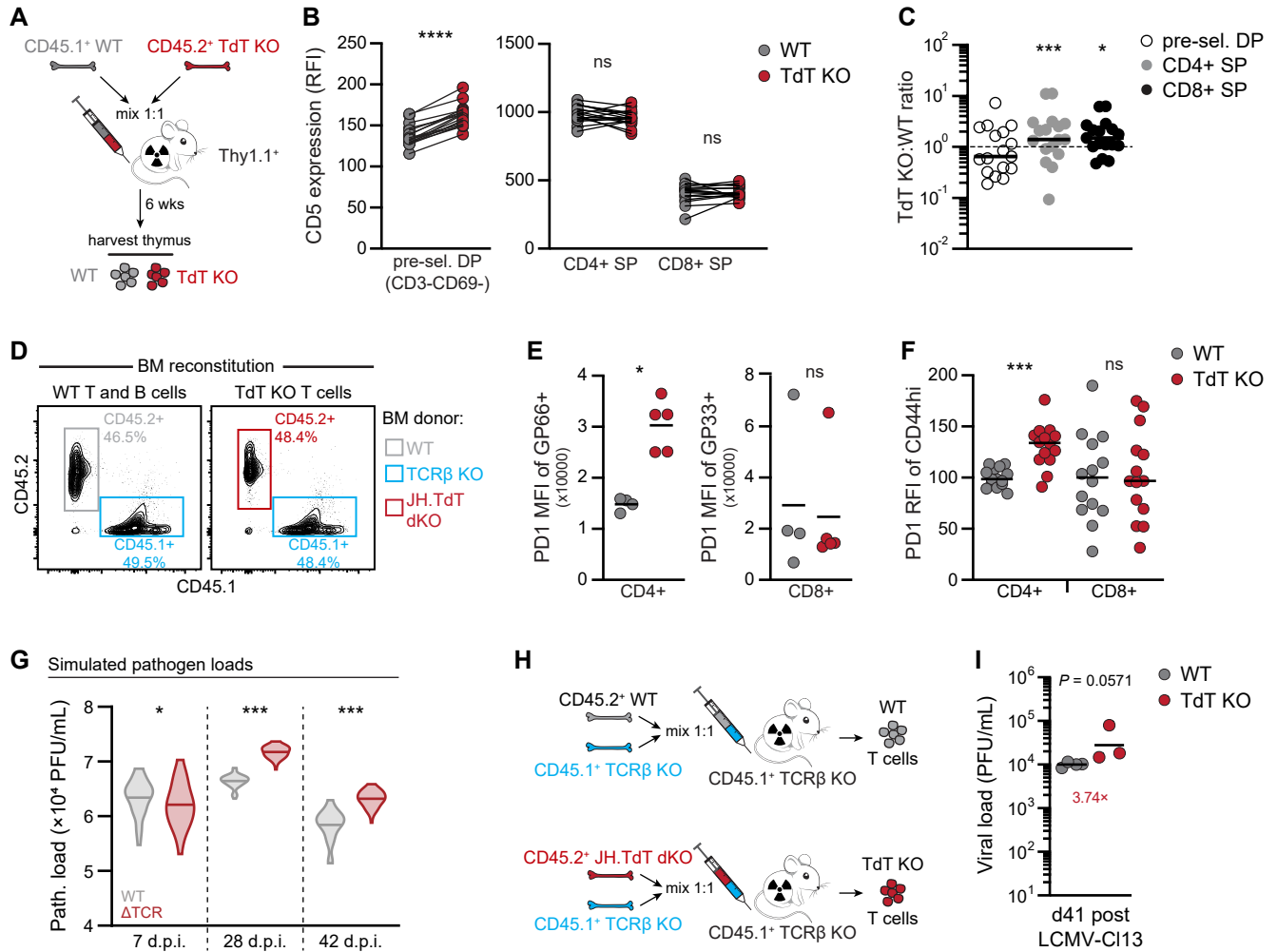

Supplement: S4 Fig — (A) Mixed BM chimeras possessing WT and TdT KO cells (reconstitution of irradiated mice with 1:1 ratio of BM cells) were generated, and the thymus was harvested 6 weeks after irradiation for each mouse. (B, C) RFI of surface CD5 expression on pre-selection DP thymocytes (left) and CD4+ and CD8+ SP thymocytes (right), relative to CD5 expression on WT double-negative thymocytes; lines connect WT and TdT KO cells from the same chimeric mouse (B). Ratio of TdT KO to WT pre-selection DP, CD4+ SP, and CD8+ SP thymocytes (C). Data are summarized from 2 independent experiments, n = 17. (D) Representative flow cytometry plots showing the percent of BM cells from each set of donor mice, namely, CD45.1+ cells from TCRβ KO mice and CD45.2+ cells from either WT or JH × TdT double KO mice. (E) Summary MFI of PD1 expression on WT and TdT KO tetramer-positive activated CD4+ (CD44hi GP66:I-Ab+) and CD8+ (CD44hi GP33:Db+) T cells, n = 4–5 chimeras. (F) RFI of surface PD1 expression on total activated (CD44hi) CD4+ and CD8+ T cells normalized to expression on activated (CD44hi) WT CD4+ or CD8+ T cells, respectively. Data are summarized from 4 independent experiments. n = 14 chimeras. (G) Pathogen loads generated from 50 model simulations at time points equivalent to those indicated in Fig 4J, using either the WT or the ΔTCR repertoire configurations (Fig 4C). Horizontal lines indicate mean values. (H) Modified experimental approach from Fig 4F by using TCRβ KO mice as irradiated BM recipients. (I) LCMV-Cl13 viral loads in the serum of recipient TCRβ KO mice reconstituted with WT or TdT KO T cells at day 41 post-infection. P values indicated were computed using Wilcoxon matched-pairs signed rank test (B), Friedman test (C), Mann–Whitney test (E and F), and two-tailed Wilcoxon rank sum test on geometric means (H). ns = not significant, * P < 0.05, *** P < 0.001. The experimental data underlying this figure can be found in S1 Data. See Methods to access code used to produce model simulat [file pbio.3002465.s004.pdf]

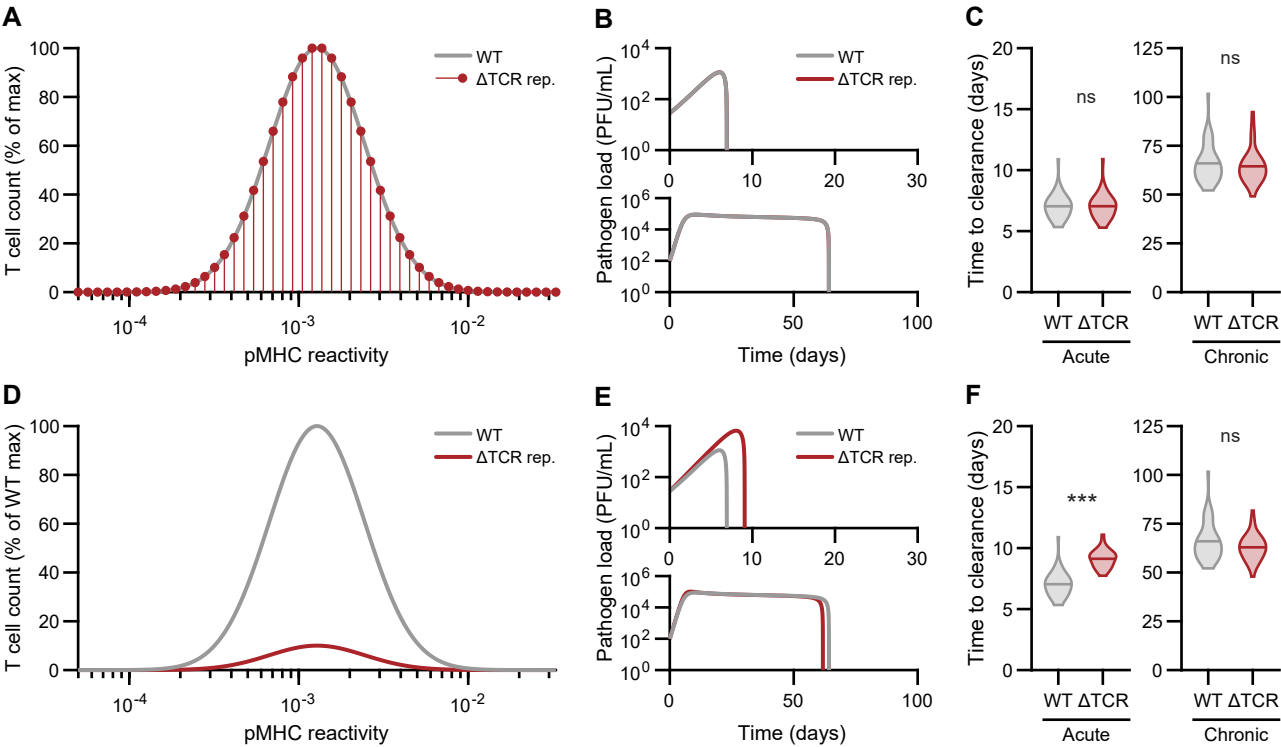

Supplement: S5 Fig — (A, D) Altered TCR repertoire obtained by either reducing the number of clonotypes by decreasing N to 50, resulting in fewer clones across the entire pMHC reactivity range while keeping the total T cell count constant (A), or reducing precursor frequency across all pMHC reactivity values by decreasing σE,tot 10-fold to 2.97 cells day−1 (D). (B, E) Model simulations comparing representative pathogen load traces of WT (gray) and ΔTCR repertoires (red) configurations in (A) and (C) during acute (top) or chronic (bottom) infection when the number of clones is reduced (B) or when precursor frequencies are reduced (E). Note the overlap of the 2 curves in (B). (C, F) Time to clearance of acute (left) or chronic (right) infections for 50 model simulations (log-values of initial pathogen loads randomized to ±10% of log-values in S1 Table) from WT and ΔTCR repertoire systems associated with reducing number of clones (C) or precursor frequencies (F). Horizontal lines indicate mean values. ns = not significant; ***, P < 0.001. P values computed using the Wilcoxon rank sum test. See Methods to access code used to produce model simulations in this figure. pMHC, peptide-major histocompatibility complex; TCR, T cell receptor; TdT, terminal deoxynucleotidyl transferase; WT, wild type. (PDF) [file pbio.3002465.s005.pdf]

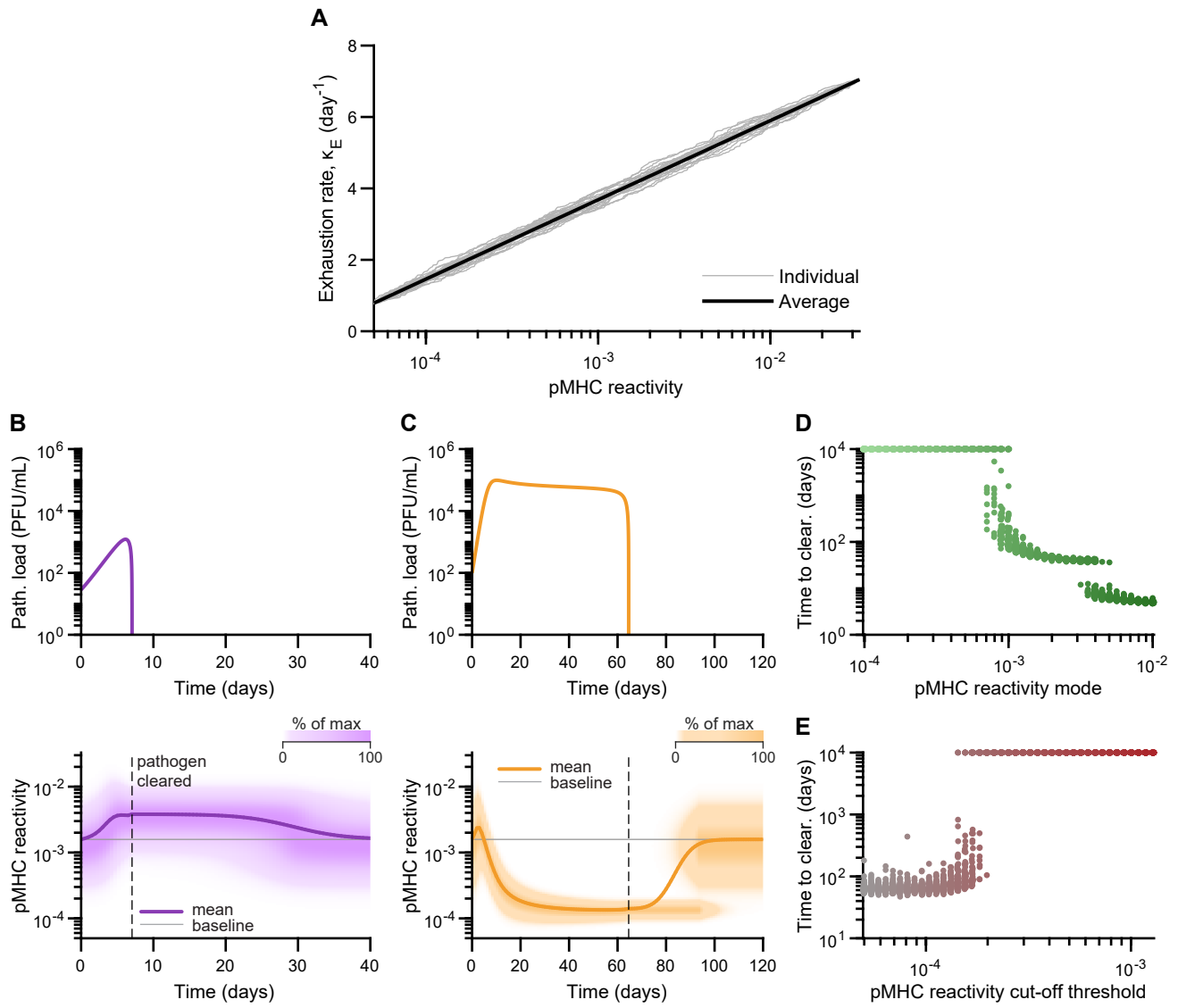

Supplement: S6 Fig — (A) Function depicting pMHC reactivity-dependent exhaustion rate, κE, when sampling from a uniform distribution (with a lower bound of κE,min as in S1 Table, and upper bound κE,max set to 6.67 day−1) and sorting in ascending order. (B, C) Pathogen load (top) and pMHC-reactivity distribution (bottom) obtained by simulating the model response to acute (B) or chronic (C) pathogen, when κE was sampled from uniform distribution. Chronic replication rate (rP) was reduced to 1.15 day−1; all other parameters were kept at their default values shown in S1 Table. (D, E) Effect of varying the pMHC-reactivity mode (D), as in Fig 3A, or of removing T cells with low pMHC-reactivity (E), as in Fig 4B, when κE was sampled from uniform distribution. Note that all results are consistent with those obtained by sampling κE from an exponential distribution as shown in Fig 1C. See Methods to access code used to produce model simulations in this figure. (PDF) [file pbio.3002465.s006.pdf]
