# Supplementary material for: Chronic infection control relies on T cells with lower foreign antigen binding strength generated by N-nucleotide diversity
Source: PLoS Biol. 2024 Feb 1;22(2):e3002465. doi: 10.1371/journal.pbio.3002465 (PMC10833529; doi:10.1371/journal.pbio.3002465)
Supplement: S1 Table — Values were determined by fitting model simulations to serum data in acute vs. chronic LCMV infection [22] using a genetic algorithm. (DOCX) [file pbio.3002465.s007.docx]

S1 Table. Parameter values used in model simulations. Values were determined by fitting model simulations to serum data in acute vs. chronic LCMV infection (1) using a genetic algorithm.

| **Param.** | **Description** | **Value(s)** | **Units** |
| --- | --- | --- | --- |
| $r_{P}$ | Maximum pathogen replication rate | 0.77 (acute)  1.22 (chronic) | day^-1^ |
| $P_{0}$ | Initial pathogen load | 28.4 (acute)  98.3 (chronic) | PFU mL^-1^ |
| $P_{\max}$ | Pathogen carrying capacity | 1.17×10^5^ | PFU mL^-1^ |
| $\kappa_{P}$ | Maximum pathogen removal rate per T cell | 4.94×10^-2^ | PFU mL^-1^ cell^-1^ day^-1^ |
| $k$ | Pathogen load at half-maximum activation of T cells | Range | PFU mL^-1^ |
| $a_{k}$ | pMHC reactivity parameter (magnitude equal to $1/k$) | Range | unitless |
| $a$ | Scaling factor of half-maximum constant in pathogen clearance | 1.77×10^-3^ | unitless |
| $\sigma_{E,\mathrm{tot}}$ | Total thymic input across all pMHC-reactivity values | 29.7 | cells day^-1^ |
| $k_{\mathrm{mode}}$ | Mode of the pMHC-reactivity function at which thymic input is maximal | 7.8×10^2^ | PFU mL^-1^_­­_ |
| $k_{\mathrm{span}}$ | Span of the pMHC-reactivity function | 1.91 | unitless |
| $r_{E}$ | Proliferation rate of effector T cells | 3.19 | day^-1^ |
| $\delta_{E}$ | Natural turnover rate of effector T cells | 0.28 | day^-1^ |
| $\kappa_{E}$ | Pathogen-dependent T cell exhaustion rate - sampled from a shifted exponential distribution $g\left( k;\mu\right)+\kappa_{E,\min}$ and sorted (highest $\alpha_{k}$ corresponds to highest $\kappa_{E}$ value) | $\mu$=3.34 | day^-1^ |
| $\kappa_{E,\min}$ | Minimum exhaustion rate | 0.39 | day^-1^ |
| $b$ | Scaling factor of half-maximum constant of T cell exhaustion | 38.1 | unitless |
| $\varepsilon$ | Inter-cellular competition rate | 3.24×10^-6^ | cell^-1^ day^-1^ |
| $N$ | Number of T-cell clones obtained from discretizing Eqs. (1)-(2) (see *Numerical simulation* section) | 500 | unitless |
