# Supplementary material for: Chronic infection control relies on T cells with lower foreign antigen binding strength generated by N-nucleotide diversity
Source: PLoS Biol. 2024 Feb 1;22(2):e3002465. doi: 10.1371/journal.pbio.3002465 (PMC10833529; doi:10.1371/journal.pbio.3002465)
Supplement: S1 Text — Description of model parameters and fitting, detailed numerical implementation, stability analysis of the full model, and analysis of a reduced, one-clone model. (DOCX) [file pbio.3002465.s011.docx]

**Supporting Information – S1 Text**

**Model parameters and fitting**

In order to generate parameter values for model simulations, a genetic algorithm was used. The fitness function of the genetic algorithm was primarily based on minimizing the sum of squared errors between the data and model predictions (i.e., minimizing $\sum_{i} \left( \boldsymbol{Y}_{i}-F\left( t;\boldsymbol{P} \right) \right)^{2}$, where $\boldsymbol{Y}_{i}$ is the serum time series data in (1), and $F(t,\boldsymbol{P})$ is the fit output as a function of time with input parameter vector $\boldsymbol{P}$). The results were then refined to ensure the final selected parameter values satisfied the following conditions:

- The pathogen replication rate for chronic LCMV (LCMV-Cl13) is larger than that for acute LCMV (LCMV-Arm), as suggested by (2, 3)
- Pathogen load starts from a perturbed initial value $P_{0}$ (a fitted parameter, see below), and the rate of change of pathogen is initially positive i.e., ${dP}/{dt}>0$ at $t=0$.
- The steady state of the pathogen-free equilibrium is stable (see “*Stability analysis”* section below).
- An upper bound for the standard deviation of the function describing the $\sigma_{E}$ distribution, $f\left( k; \mu,\sigma\right)$, is applied to ensure that $f$ is negligibly small for non-physiologically small values of $k$ (i.e., for pMHC reactivities that are too high).

To specify the lower and upper bounds used by the parameter sampling in the genetic algorithm, ranges from similar parameters in the effector T cell dynamics (Eq. 2 in the main text) from previous mathematical models were used (4-6). In order to fit serum viral loads digitized from (1), we used the early time points to inform our choices for the bounds on the replication rate, $r_{P}$, and the initial pathogen load, $P_{0}$, in the context of the LCMV-Cl13 data. This was done by assuming that T cell influence is negligible early in infection, and thus pathogen expansion during this phase of the infection to be predominantly dictated by exponential growth. In other words, we assumed that pathogen load dynamics (Eq. 1 in the main text) are governed by

$$\frac{dP}{dt}\approx r_{P}P,$$

for $P\ll P_{\max}$, where $P_{\text{max}}$ is the pathogen carrying capacity. Solving this latter equation, we obtained an approximation for $P(t)$ early in the infection, given by

$$P\left( t \right)\approx P_{0}\exp(r_{P}t)$$

or

$\ln P(t)\approx r_{P}t+\ln P_{0}$,

where $P_{0}=P(t=0)$. Fitting this linear expression of $\ln P\left( t \right)$ in terms of $t$ to the digitized data in (1), we obtained an estimate for the confidence bounds on $r_{P}$ and $\ln P_{0}$. The range used for the pathogen carrying capacity, $P_{\max}$, was set to be on the order of ${10}^{5}$ PFU/mL, a value that is consistent with the range of values of LCMV-Cl13 in the serum at peak infection in the data.

The pMHC-reactivity distribution mode and span, parameters that are not identifiable but important for the study, were sampled from wide ranges by the genetic algorithm. In the case of the mode, it was sampled uniformly on a log scale, unlike the other parameters that are sampled uniformly on a linear scale. To define the distribution of thymic input defined by the log-normal function of pMHC reactivity, we let $\sigma_{E}=\sigma_{E,\mathrm{tot}} f\left( k;\mu,\sigma\right)$, where $f$ is the log-normal probability density function with mean and standard deviation $\mu$ and $\sigma$, respectively, and $\sigma_{E,\mathrm{tot}}$ is the total thymic input of pathogen-specific T cells across all pMHC-reactivity values. The mode of the $a_{k}$ distributions used in **Fig 3A** is given by$1/{\exp\left( \mu+\sigma^{2} \right)}$, where $a_{k}=1/k$, and the span of these distributions used in **Fig 3B** is $\exp(\sigma)$.

Let $k_{\mathrm{mode}}=\exp\left( \mu+\sigma^{2} \right)$ and $k_{\mathrm{span}}=\exp\sigma$ be the mode and span, respectively, that define the log-normal probability density function $f$. Since these quantities are simpler to think about than $\mu$ and $\sigma$ themselves, we defined an equivalent function $\sigma_{E}=\sigma_{E,\mathrm{tot}} \tilde{f}\left( k;k_{\mathrm{mode}},k_{\mathrm{span}} \right)$. The parameters $k_{\mathrm{mode}}$ and $k_{\mathrm{span}}$ are the parameters that were fit by the genetic algorithm. We assigned the upper and lower bounds on $k$ ($k_{\max}$, and $k_{\min}$, respectively) to be at $\pm5\sigma$ in log space from the fitted mode $k_{\mathrm{mode}}$.

**Numerical simulation**

To simulate the system of integro-differential equations, we discretized the continuous T cell population $E(t,k)$, into individual clones of T cells $E_{i}$ with pMHC reactivity $a_{k,i}={1/k}_{i}$, where $i=1,2,\ldots,N$ and $N$ is the number of T cell clonotypes. To approximate a pMHC-reactivity continuum, we chose a large value for $N$. The resulting model becomes a high-dimensional system of ordinary differential equations with $N+1$ variables, whose equations can be expressed as

$$\frac{dP}{dt}=r_{P} P \left( 1-\frac{P}{P_{\max}} \right)-\kappa_{P} \sum_{i=1}^{N} E_{i}\frac{P}{P+a k_{i}}$$

$$\frac{dE_{i}}{dt}=\sigma_{E}(k_{i})+r_{E} E_{i} \frac{P}{P+k_{i}}-\delta_{E} E_{i}-\kappa_{E}(k_{i}) E_{i} \frac{P}{P+b k_{i}}- \varepsilon E_{i}\sum_{j=1}^{N} E_{j}.$$

To initialize the simulations, we first allowed $E_{i}$ to evolve toward their baseline values in the absence of pathogen, i.e., when $P=0$ as described in the next section. Then, we introduced a non-zero perturbation to the pathogen load at time $t=0$, i.e., $P\left( t=0 \right)\to P_{0}$ where $P_{0}$ is obtained from parameter fitting as described previously.

**Stability analysis of the full model**

One can show that $\left( P,\boldsymbol{E} \right)=(0,\boldsymbol{E}^{\boldsymbol{*}}\boldsymbol{)}$, where $\boldsymbol{E}^{\boldsymbol{*}}\boldsymbol{=(}E_{1}^{*}\boldsymbol{,}E_{1}^{*}\boldsymbol{,}\ldots\boldsymbol{,}E_{N}^{*}\boldsymbol{)}$ is the level of each effector T cell clone in the pathogen-free equilibrium, is always a steady state solution of the full, discretized system. We denoted this steady state by $\boldsymbol{S}_{\boldsymbol{0}}$, which represents the state of the system at homeostasis, i.e., in the absence of pathogen. Since we did not explicitly incorporate a memory formalism into the mathematical model, $\boldsymbol{S}_{\boldsymbol{0}}$ is both the baseline state that effector T cells are initialized from prior to the introduction of pathogen, as well as the state that the system evolves to upon pathogen clearance. Note that $\boldsymbol{S}_{\boldsymbol{0}}$ is distinct from the initial state of the model upon infection, which begins at $\left( P,\boldsymbol{E} \right)=(P_{0},\boldsymbol{E}^{\boldsymbol{*}}\boldsymbol{)}$**,** as described above. In this section, we will determine the condition needed to ensure that $\boldsymbol{S}_{\boldsymbol{0}}$ is stable. This condition is then imposed on the genetic algorithm when evaluating parameter fitness.

The Jacobian matrix of the full system evaluated at the steady state $\boldsymbol{S}_{\boldsymbol{0}}=(0,\boldsymbol{E}^{\boldsymbol{*}}\boldsymbol{)}$ can be written as

$$\mathbf{J}_{\boldsymbol{S}_{0}}=\left( \begin{matrix} r_{P}-\frac{\kappa_{P}}{a}\sum_{n=1}^{N} \frac{E_{n}^{*}}{k_{n}} & 0 & 0 & \cdots& 0 \\ \frac{E_{1}^{*}}{k_{1}}\left( r_{E}-\frac{\kappa_{E}}{b} \right) & -\delta_{E}-\varepsilon\sum_{n=1}^{N} E_{n}^{*}-\varepsilon E_{1}^{*} & -\varepsilon E_{1}^{*} & \cdots& -\varepsilon E_{1}^{*} \\ \frac{E_{2}^{*}}{k_{2}}\left( r_{E}-\frac{\kappa_{E}}{b} \right) & -\varepsilon E_{2}^{*} & -\delta_{E}-\varepsilon\sum_{n=1}^{N} E_{n}^{*}-\varepsilon E_{2}^{*} & \cdots& -\varepsilon E_{2}^{*} \\ \vdots& \vdots& \vdots& \ddots& \vdots\\ \frac{E_{N}^{*}}{k_{N}}\left( r_{E}-\frac{\kappa_{E}}{b} \right) & -\varepsilon E_{N}^{*} & -\varepsilon E_{N}^{*} & \cdots& -\delta_{E}-\varepsilon\sum_{n=1}^{N} E_{n}^{*}-\varepsilon E_{N}^{*} \end{matrix} \right).$$

The eigenvalues of this Jacobian matrix are given by

$$\lambda_{0}=r_{P}-\frac{\kappa_{P}}{a}\sum_{n=1}^{N} \frac{E_{n}^{*}}{k_{n}} , \lambda_{1}=-\delta_{E}-2\varepsilon\sum_{n=1}^{N} E_{n}^{*}, \lambda_{i}=-\delta_{E}-\varepsilon\sum_{n=1}^{N} E_{n}^{*}, i=\left\{ 2, 3,\ldots,N \right\}.$$

It follows that $\lambda_{0}<0$is a necessary and sufficient condition to ensure local stability of $\boldsymbol{S}_{\boldsymbol{0}}$, as all other eigenvalues are always negative for positive parameter values and T-cell levels. This condition may be rewritten as

$$\sum_{n=1}^{N} \frac{E_{n}^{*}}{k_{n}}>\frac{ar_{P}}{\kappa_{P}}.$$

The individual values of $E_{n}^{*}$ may be computed by simulating the full model in the absence of pathogen (i.e., for $P=0$); this condition can then be verified during the parameter fitness evaluation process of the genetic algorithm to reject parameter selections that do not ensure the stability of $\boldsymbol{S}_{\boldsymbol{0}}$.

The existence and stability of other steady states in the model depend on different parameter values. To assess them, we employed a bifurcation analysis approach applied on a simplified single-clone ($N=1$), 2-dimensional model.

**Analysis of the reduced one-clone, 2D model**

To better understand the underlying dynamics of the full continuum model and the distinct time scales between outcomes of an acute and chronic infection, we turned our attention to a simplified, single-clone version of the model (with $N=1$), given by

$$\frac{dP}{dt}=r_{P} P \left( 1-\frac{P}{P_{\max}} \right)-\kappa_{P} E\frac{P}{P+a k}$$

$$\frac{dE}{dt}=\sigma_{E}+r_{E} E \frac{P}{P+k}-\delta_{E} E-\kappa_{E} E \frac{P}{P+b k}- \varepsilon E^{2}.$$

Model parameters were assigned the same values as those provided in Table S1, except for $\sigma_{E}$, $\kappa_{E}$ and $k$. In this case, $\sigma_{E}$ was set to be $29.7$ cells/day, $\kappa_{E}$ to be $2.78$ day^-1^ and $k$ to be a bifurcation parameter spanning a wide range.

**S2A Fig** plots the steady-state levels of the pathogen load, $P$, on a log-scale, as a function of the pMHC reactivity ($\alpha_{k})$. The pathogen load was shifted by $1$, to show the behaviour at $P=0$ on a log-scale (i.e.,${10}^{0}$ corresponds to $P=0$). The figure shows that pathogen load can attain two different values at steady state (solid lines): an elevated level (hereafter referred to as the $\boldsymbol{S}_{\boldsymbol{1}}$ state) and a low level ($\boldsymbol{S}_{\boldsymbol{0}}$), both acting as attractors (as opposed to repellers shown as dashed lines) that can co-exist at high $\alpha_{k}$. This coexistence of the two attractors is a hallmark of bistability, which highlights the dependence of the system on the initial level of $P$ to determine which attractor will be eventually approached over time. For the $\boldsymbol{S}_{\boldsymbol{0}}$ steady state to be an attractor (stable), the condition $\alpha_{k}> 4.15\times{10}^{-4}$ must be satisfied.^^[[1]](#footnote-2)^^

We next showed the behaviour of the system with respect to the pathogen-dependent effector T-cell exhaustion rate, $\kappa_{E}$ (**S2B Fig**). Pathogen loads can be either elevated or zero due to the presence of bistability for $\kappa_{E}>0.84$ day^-1^. Below this critical value of $\kappa_{E}$, only the lower steady state exists as the system’s global attractor.^^[[2]](#footnote-3)^^

To understand the effects of such dynamics of the single-clone model on the full, continuum model, we computed the evolution of the weighted average of pMHC reactivity and that of the exhaustion rate of dominant effector T cells throughout the chronic immune response and overlayed this trajectory on the 2-parameter bifurcation diagram of viral load with respect to $\kappa_{E}$ and $\alpha_{k}$ of the single clone model (**S2C Fig**). The gray-shaded region represents the bistable region in **S2B Fig**. The starting values of the average exhaustion rates and pMHC reactivities fall within the bistable region; acute vs. chronic outcomes are set apart by whether the system evolves immediately toward $\boldsymbol{S}_{\boldsymbol{0}}$ (acute), or if it first goes to the upper $\boldsymbol{S}_{\boldsymbol{1}}$ state and can only come back down once the full system moves out of the gray region. The latter, as a result, takes much longer, causing the full model to produce the time scale separation (clustering) in the time to clearance between acute vs. chronic infections.

This time scale separation observed in the full model can be further visualized by examining how the nullclines of the one-clone system dictate the dynamics of the full model when the average values of $a_{k}$ and $\kappa_{E}$ parameters change; this was done by superimposing a solution trajectory of the full model on the phase space of the one-clone system and observing how they all evolve over time (**S2 Movie**). Doing so revealed that, during a chronic infection, the trajectory evolves toward a transiently existing stable steady state with elevated pathogen load (i.e., toward $\boldsymbol{S}_{\boldsymbol{1}}$). The fixed point $\boldsymbol{S}_{\boldsymbol{1}}$, however, eventually disappears at a saddle-node bifurcation as the average pMHC reactivity and exhaustion rate parameters of the full model decrease, leading the solution trajectory to return back to $\boldsymbol{S}_{\boldsymbol{0}}$ (where the $E$-nullcline meets the vertical $P$-nullcine). Note that the initial state of the simulations from which the numerical solution is computed, i.e., the small perturbation $P_{0}$ located horizontally to the right of $\boldsymbol{S}_{\boldsymbol{0}}$, falls under the parabolic $P$-nullcline, allowing the pathogen to grow initially. Whether the solution follows a small loop before returning to $\boldsymbol{S}_{\boldsymbol{0}}$ (acute), or evolves toward the transiently existing $\boldsymbol{S}_{\boldsymbol{1}}$ (chronic), depends on whether the initial condition lies to the left or to the right, respectively, of the saddle fixed point’s stable manifold.

Of note, the equations representing effector T cell dynamics, and the resulting bifurcation diagram, bear resemblance to the model studying post-treatment control of HIV-1 infection described in (7). In that study, bistability was also an important feature of the model, explaining how some individuals infected with HIV-1 could maintain undetectable viral loads after cessation of anti-retroviral treatment. However, our model differs in that we do not observe a third stable state with non-zero albeit undetectable low pathogen loads.

**SI References**

1. E. J. Wherry, J. N. Blattman, K. Murali-Krishna, R. Van Der Most, R. Ahmed, Viral persistence alters CD8 T-cell immunodominance and tissue distribution and results in distinct stages of functional impairment. *Journal of Virology* **77**, 4911-4927 (2003).

2. A. Bergthaler *et al.*, Viral replicative capacity is the primary determinant of lymphocytic choriomeningitis virus persistence and immunosuppression. *Proceedings of the National Academy of Sciences* **107**, 21641-21646 (2010).

3. B. M. Sullivan *et al.*, Point mutation in the glycoprotein of lymphocytic choriomeningitis virus is necessary for receptor binding, dendritic cell infection, and long-term persistence. *Proceedings of the National Academy of Sciences* **108**, 2969-2974 (2011).

4. A. Khadra, P. Santamaria, L. Edelstein-Keshet, The role of low avidity T cells in the protection against type 1 diabetes: a modeling investigation. *Journal of Theoretical Biology* **256**, 126-141 (2009).

5. M. Jaberi-Douraki, M. Pietropaolo, A. Khadra, Continuum model of T-cell avidity: understanding autoreactive and regulatory T-cell responses in type 1 diabetes. *Journal of Theoretical Biology* **383**, 93-105 (2015).

6. H. Jamaleddine, P. Santamaria, A. Khadra, Quantifying immunoregulation by autoantigen‐specific T‐regulatory type 1 cells in mice with simultaneous hepatic and extra‐hepatic autoimmune disorders. *Immunology* **161**, 209-229 (2020).

7. J. M. Conway, A. S. Perelson, Post-treatment control of HIV infection. *Proceedings of the National Academy of Sciences* **112**, 5467-5472 (2015).

1. Note that units are omitted from the discussion of the pMHC-reactivity measure, since $\alpha_{k}$ does not represent a direct value for the affinity of the T cell receptor but rather acts as an indicator for it. [↑](#footnote-ref-2)
2. Within the physiological range of the system, $P\geq0$ and $E\geq0$. [↑](#footnote-ref-3)
